# Biomimetic mineralization of metal-organic frameworks as protective coatings for biomacromolecules

**DOI:** 10.1038/ncomms8240

**Published:** 2015-06-04

**Authors:** Kang Liang, Raffaele Ricco, Cara M. Doherty, Mark J. Styles, Stephen Bell, Nigel Kirby, Stephen Mudie, David Haylock, Anita J. Hill, Christian J. Doonan, Paolo Falcaro

**Affiliations:** 1 CSIRO Manufacturing Flagship, Private Bag 10, Clayton South, Victoria 3169, Australia; 2 Department of Chemistry, The University of Adelaide, Adelaide, South Australia 5005, Australia; 3 Australian Synchrotron, 800 Blackburn Road, Clayton, Victoria 3169, Australia; 4 The Australian Regenerative Medicine Institute, Monash University, Wellington Road, Clayton, Victoria 3800, Australia

## Abstract

Enhancing the robustness of functional biomacromolecules is a critical challenge in biotechnology, which if addressed would enhance their use in pharmaceuticals, chemical processing and biostorage. Here we report a novel method, inspired by natural biomineralization processes, which provides unprecedented protection of biomacromolecules by encapsulating them within a class of porous materials termed metal-organic frameworks. We show that proteins, enzymes and DNA rapidly induce the formation of protective metal-organic framework coatings under physiological conditions by concentrating the framework building blocks and facilitating crystallization around the biomacromolecules. The resulting biocomposite is stable under conditions that would normally decompose many biological macromolecules. For example, urease and horseradish peroxidase protected within a metal-organic framework shell are found to retain bioactivity after being treated at 80 °C and boiled in dimethylformamide (153 °C), respectively. This rapid, low-cost biomimetic mineralization process gives rise to new possibilities for the exploitation of biomacromolecules.

## Abstract (web summary)

Robust biomacromolecules could be used for a wide range of biotechnological applications. Here the authors report a biomimetic mineralization process, in which biomolecules are encapsulated within metal-organic frameworks, and their stability is subsequently increased without significant bioactivity loss.

## Graphical abstract

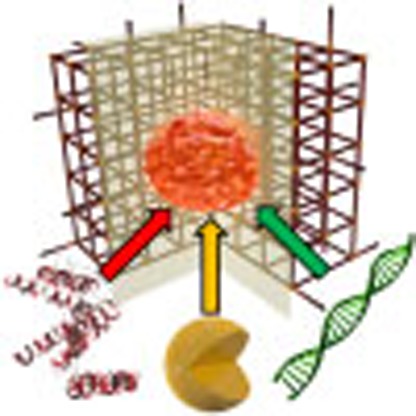

Many living organisms fabricate molecular architectures specifically designed to provide exoskeletal protection and structural support for soft tissue^1^. This biologically induced, self-assembly process, termed biomineralization, is carried out with exquisite control of crystal morphology and compositional specificity under physiological conditions^2^. Furthermore, the resultant biocomposites typically exhibit superior mechanical properties with respect to their constituents^3^. Accordingly, such natural biomineralization processes have inspired ‘biomimetic'^4^ strategies for the synthesis of novel materials for photonics, biomedical implants, drug delivery and biochemical separations^5^,^6^,^7^,^8^.

A goal of biomimetic mineralization is to adopt and translate the self-assembly processes found in natural biological systems to the development of a general method for encapsulating bioactive molecules within protective exteriors^9^. Success in this endeavour would significantly increase the potential for utilizing functional biomacromolecules in applications where enhanced thermal stability, tolerance to organic solvents or extended shelf-lifetime is required, such as industrial catalysis and biopharmaceutical delivery^10^,^11^,^12^. To this end, recent studies have demonstrated that coating bioactive macromolecules with inorganic shells (for example, calcium phosphate, CaP) can provide prolonged shelf-lifetime^9^. Although this report clearly demonstrates the potential applications of exploiting biomineral shells, seeding an inorganic architecture at an organic interface presents a significant challenge. Indeed, to stimulate growth of a CaP shell on vaccines, genetic modification was required to incorporate a specific peptide sequence known to have a high affinity for calcium ions^9^. This result highlights that enhancing the interfacial interactions between inorganic and organic components is a key to inducing biomineralization^13^. However, given that the tertiary structure of many biomacromolecules is critical to their function, it is unlikely that a strategy that involves modifying the primary peptide sequence could be generally applied. We posit that hybrid organic–inorganic materials would provide a more versatile and general method for encapsulating biomacromolecules as, in general, the protein domains have a high affinity for the organic moieties^14^. A rapidly growing class of hybrid materials termed metal-organic frameworks (MOFs) represent excellent candidates for this purpose, as they are: constructed from organic and inorganic components^15^, thermally and chemically stable^16^,^17^,^18^,^19^, can be grown on different substrates (films^20^, particles^21^ and gels^22^), and under mild biocompatible conditions^19^. In addition, they have been utilized as a biocompatible material for drug release^19^,^23^. Furthermore, MOFs possess open architectures and large pore volumes^24^, which can facilitate the selective transport of small molecules through the protective porous coating^25^,^26^, enabling the selective interaction of the biomacromolecule (for example, enzymes) with the external environment. Hitherto, attempts to integrate biomacromolecules within MOFs have been limited in application by two main constraints: for post-synthetic infiltration only biomacromolecules analogous in size to the MOF pores can be loaded into the framework^27^,^28^,^29^, and in the preparation of MOF biocomposites only solvent resistant biomacromolecules can be used^30^. Overcoming these limitations would facilitate the fabrication of novel biocomposites and their exploitation for bioapplications^31^.

Here we report that a wide range of biomacromolecules including proteins, DNA and enzymes can efficiently induce MOF formation and control the morphology of the resultant porous crystal via a biomimetic mineralization process under physiological conditions. Although biomineralization has been extensively investigated for inorganic systems, the concept has not been applied to MOFs. Here we demonstrate that the biomimetic mineralization of MOFs forms a nanoporous shell, which encapsulates the biomacromolecules and affords unprecedented protection from biological, thermal and chemical degradation with maintenance of bioactivity (Fig. 1). Furthermore, the encapsulated biomacromolecules can be released simply by a pH change within a physiological environment. Zeolitic imidazolate framework-8 (ZIF-8), formed by coordination between Zn^2+^ ions and 2-methylimidazole (HmIm)^32^, is selected as a candidate MOF material for this study due to its high surface area, exceptional chemical and thermal stability and negligible cytotoxicity^23^,^32^,^33^. In addition, we demonstrate that other MOFs (HKUST-1, Eu/Tb-BDC and MIL-88A) can also be formed, highlighting the versatility of this biomimetic mineralization approach.

## Results

### Biomimetic mineralization using bovine serum albumin

**Figure 2:**
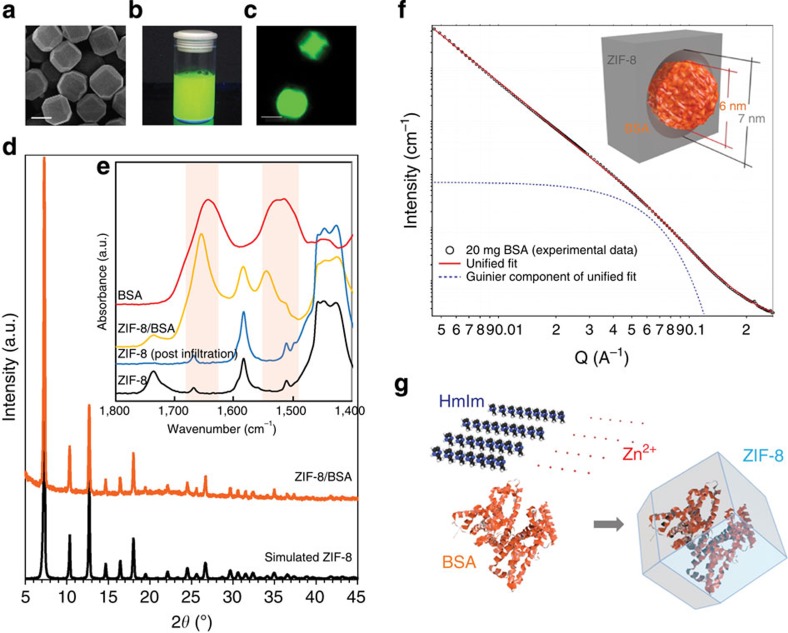
Characterization of biomimetically mineralized biocomposite. (**a**) SEM image showing the crystals obtained using BSA as a growth agent for biomimetic mineralization (scale bar, 1 μm). (**b**) Photograph and (**c**) confocal laser scanning microscopy image of the biomomimetically mineralized ZIF-8 composite obtained using BSA labelled with FITC. This biocomposite (ZIF-8/FITC-BSA) was prepared at 37 °C, washed and exposed to ultraviolet light of wavelength 365 and 495 nm, respectively (scale bar, 10 μm). (**d**) PXRD of the MOF-BSA biocomposite. (**e**) FTIR spectra of BSA (red), ZIF-8/BSA (orange), standard ZIF-8 post incubated with BSA after washing (blue), and standard ZIF-8 (black). (**f**) SAXS data of the ZIF-8/BSA biocomposite and a schematic showing the relative size of BSA to the mesopore. The observed Guinier knee can be fitted using the Unified model^58^ with a radius of gyration (*R*_g_)^59^ of 35 (± 5) Å, which is 17% larger than that of BSA 29.9 Å (ref. 60). (**g**) Schematic proposing the biomimetically mineralized growth of ZIF-8. Each BSA molecule attracts 31 2-methylimidazole (HmIm) ligands and 22 Zn^2+^ ions, facilitating the nucleation of ZIF-8 crystals^13^.

In a typical experiment for the biomimetically mineralized growth of ZIF-8, an aqueous solution containing HmIm, (160 mM, 2 ml) and bovine serum albumin (BSA, 1 mg) were mixed with a separate aqueous solution of zinc acetate (40 mM, 2 ml) at room temperature. The solution instantaneously turned from transparent to opaque (within 1 s, Supplementary Fig. 1). After centrifugation and washing, truncated cubic crystals of ZIF-8, (∼1 μm) were found by scanning electron microscope (SEM; Fig. 2a) and powder X-ray diffraction (PXRD) measurements (Fig. 2d, Supplementary Methods). To ascertain that BSA is indeed encapsulated by a ZIF-8 crystalline shell, solutions of biomimetically mineralized ZIF-8 that had been washed with a surfactant to remove any surface bound proteins were examined by Fourier transform infrared spectroscopy (FTIR). The resulting spectra showed stretches characteristic of the BSA protein at ∼1,640–1,660 and 1,510–1,560 cm^−1^, corresponding to amide I (mainly from C=O stretching mode) and II band (mainly from a combination between of NH bending and CN stretching modes), respectively^34^. However, FTIR measurements collected from samples prepared by washing a solution containing a mixture of pre-formed ZIF-8 crystals and BSA protein with surfactant did not afford stretches attributable to BSA (Fig. 2e). These data clearly demonstrate that the mechanism of BSA encapsulation is not via absorption nor adsorption through the ZIF-8 pore network. Additional evidence of biomacromolecule encapsulation was garnered by using BSA tagged with fluorescein isothiocyanate (FITC) to induce the formation of ZIF-8. The resultant biocomposite was washed with surfactant and exposed to ultraviolet light, whereon a green emission at 521 nm characteristic of FITC was observed (Fig. 2b). Furthermore, confocal microscopy revealed that the cross-sections of ZIF-8/BSA crystals were homogenously luminescent (Fig. 2c, details in Supplementary Figs 2 and 3). In contrast, crystals isolated from a mixture of pre-formed ZIF-8 and FITC-labelled BSA were not emissive (Supplementary Fig. 4).

### Physical characterization of the ZIF-8/BSA biocomposite

To assess the permanent porosity of the ZIF-8/BSA biocomposite, a 77 K nitrogen adsorption isotherm was measured (Supplementary Figs 5 and 6). The resulting isotherm is best described as Type 1 in shape and BET analysis of the data afforded a surface area of 1,381 m^2^ g^−1^, showing a decrease of porosity relative to pure ZIF-8 crystals^32^, which is consistent with the presence of BSA. To further investigate the hierarchical pore structure of the ZIF-8/BSA biocomposite, small-angle X-ray scattering experiments (SAXS) were carried out. Analysis of the data indicates the presence of mesopores within the MOF with a radius of 3.5±0.5 nm (Fig. 2f). We note that such mesopores, which are of sufficient size to accommodate the biomolecule (Fig. 2f, inset), are not detected in pure ZIF-8 (Supplementary Fig. 7).

The biomineralization mechanism occurring in natural processes is widely attributed to the specific ability of amino acids, peptide fragments and more complex biological entities, to concentrate inorganic cations (for example Ca^2+^ and Zn^2+^) to seed biominerals^9^,^35^,^36^. In this case, we posit that MOF biomimetic mineralization is facilitated by the biomacromolecules affinity towards the imidazole-containing building block arising from intermolecular hydrogen bonding and hydrophobic interactions^37^,^38^. ^1^H-NMR spectroscopy and ICP analysis confirmed that each BSA protein molecule accumulates ∼31 HmIm ligands and 22 Zn^2+^ ions, respectively (Fig. 2g and Supplementary Fig. 8). Increasing the local concentration of both metal cations and organic ligands would facilitate prenucleation clusters of ZIF-8 around the biomacromolecules, and thus lead to controlled crystal formation^39^. This hypothesis is further confirmed by *in situ* synchrotron SAXS experiments designed to follow the MOF biomimetic mineralization process (Supplementary Figs 9 and 10). Analysis of the X-ray scattering reveals that small particles (radius of gyration, *R*_g_=35 nm) were formed in small quantity in the aqueous solution immediately after injection of the ZIF-8 precursors. In contrast, when BSA was introduced into the solution, the rapid formation of a second generation of larger particles (∼30 s, *R*_g_=100 nm) and a simultaneous depletion of the small particles were observed (Supplementary Figs 9 and 10). Increasing the amount of BSA added to the MOF precursor solution still facilitated the prompt formation of particles (Supplementary Figs 11–14). After separation and washing, samples of the crystalline product analysed by PXRD revealed that increasing the amount of BSA was enough to modify the ZIF-8 framework from a crystalline to mostly amorphous morphology (Supplementary Fig. 13). This further demonstrates that the described method mimics the natural processes in which the degree of crystallinity is carefully tuned by living organisms due to complex biological regulation^8^,^40^.

### Extension of bimimetic mineralization to other MOFs

Although we demonstrate biomimetically mineralized growth of ZIF-8, other types of MOFs such as Cu_3_(BTC)_2_ (HKUST-1), Eu_2_(1,4-BDC)_3_(H_2_O)_4_, Tb_2_(1,4-BDC)_3_(H_2_O)_4_ and Fe(III) dicarboxylate MOF (MIL-88A) can be successfully induced by BSA (Supplementary Figs 15–19). FTIR performed before and after the BSA-induced MOF coating shows that the amide vibrational mode for the MOF/BSA composites is shifted towards higher wavenumbers (Fig. 2e and Supplementary Fig. 20), indicating that there is a protein–MOF interaction due to the coordination between the Zn cations and the carbonyl group of the proteins^41^.

### Biomimetic mineralization extended to enzymes and DNA

**Figure 3:**
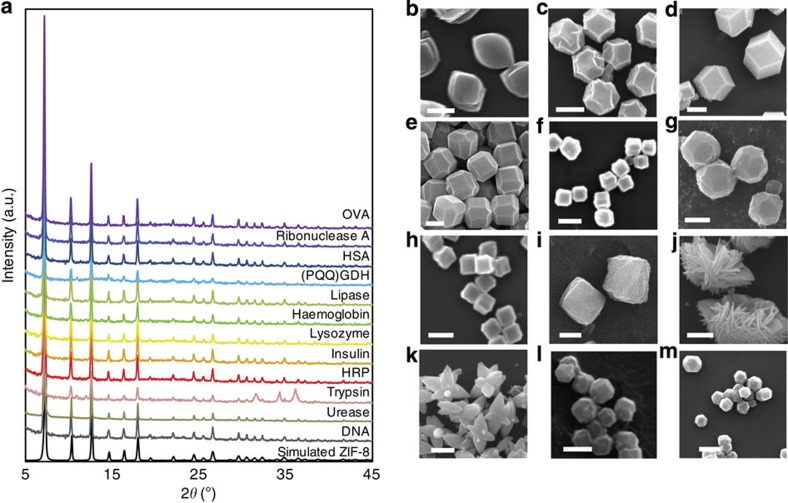
Characterization of biomimetically mineralized ZIF-8 with biomolecules. (**a**) PXRD patterns of the crystals obtained using various biomacromolecules as biomimetic mineralizion agents. Protein encapsulation efficiency: BSA ∼100%, human serum albumin (HSA) ∼100%, OVA ∼100%, lysozyme ∼96%, HRP ∼100%, ribonuclease A ∼86%, haemoglobin ∼90%, trypsin ∼96%, lipase ∼88%, insulin ∼86%, glucose dehydrogenase (PQQ-GDH) ∼82%, urease ∼95%. In each case, the intensity and peak positions of the biocomposites match those of pure ZIF-8. (**b**–**m**) Scanning electron microscopy images showing crystals obtained using: (**b**) OVA, (**c**) ribonuclease A, (**d**) HSA, (**e**) pyroloquinoline quinone-dependent glucose dehydrogenase ((PQQ)GDH), (**f**) lipase, (**g**) haemoglobin, (**h**) lysozyme, (**i**) insulin, (**j**) HRP, (**k**) trypsin, (**l**) urease and (**m**) oligonucleotide. Scale bars, 1 μm. While several biomacromolecules induce the standard rhombic dodecahedral morphology for the ZIF-8 biocomposites (**c**,**d**,**m**), other biomacromolecules gave rise to various morphological features such as truncated cubic (**e**,**f**,**h**,**i**,**l**), nanoleaf (**b**), nanoflower (**j**) and nanostar (**k**), respectively.

We sought to explore the scope of biomimetically mineralized MOF growth by using a series of biomacromolecules including ovalbumin (OVA), ribonuclease A, human serum albumin (HSA), pyrroloquinoline quinone-dependent glucose dehydrogenase ((PQQ)GDH), lipase, haemoglobin, lysozyme, insulin, horseradish peroxidase (HRP), trypsin, urease and oligonucleotide. Notably, PXRD measurements (Fig. 3a) and nitrogen gas adsorption isotherms (Supplementary Fig. 21) confirm that ZIF-8 is present in its crystalline form and remains permanently porous. Furthermore, SEM images show that the crystal morphology has a unique dependence on the biomacromolecule (Fig. 3b–m). This observation is analogous to natural biomineralization processes where intricate crystalline morphologies are common^6^. Indeed, in this work, nanoleaves, nanoflowers and nanostars are obtained by using OVA, HRP and trypsin as biomimetic mineralization agents and for single-strand DNA the typical rhombic ZIF-8 dodecahedron crystal morphology was observed.

### Protective properties of biomimetically mineralized ZIF-8

In nature, biomineral coatings are commonly used to protect soft tissue from its surrounding environment. This knowledge inspired us to examine whether MOF coatings could provide a similar barrier that would enable embedded biomacromolecules to withstand extreme conditions (for example, high temperature and organic solvents) that would normally lead to decomposition. Enhancing the robustness of functional biomacromolecules would extend their potential for use in the pharmaceutical and chemical synthesis sectors, addressing a major challenge in biotechnology^2^,^27^,^42^.

**Figure 4:**
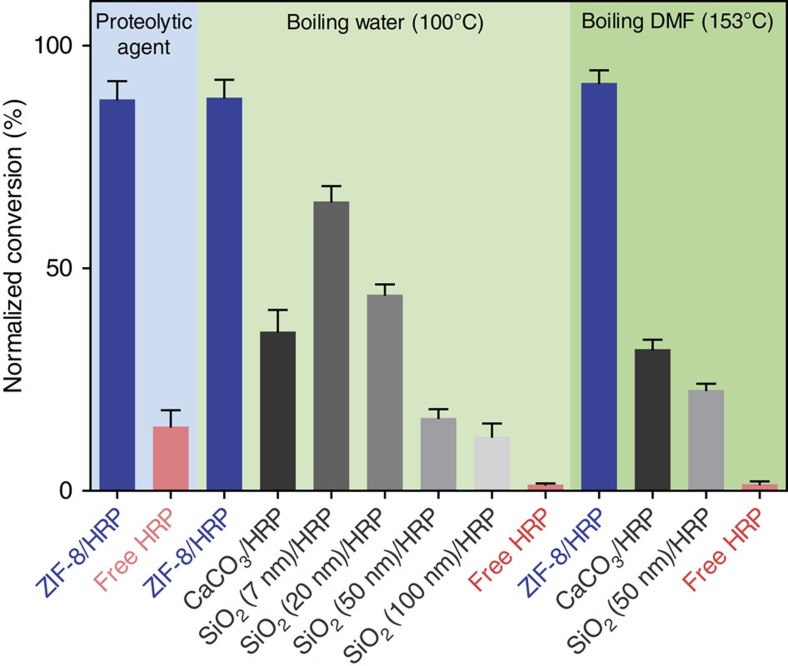
Protective performance of ZIF-8 coatings on HRP. Product conversion of free HRP, the biomimetically mineralized ZIF-8 using HRP (ZIF-8/HRP), HRP protected by calcium carbonate (CaCO_3_/HRP) and HRP protected by mesoporous silica (SiO_2_/HRP, SiO_2_ with average pore size of 7, 20, 50 and 100 nm) in the presence of proteolytic agent, trypsin, after treatment in boiling water for 1 h, and after treatment in boiling dimethylformamide (DMF) for 1 h at 153 °C, respectively. Data were normalized against free HRP activity at room temperature. Error bars represent the s.d. of three independent experiments.

Typically, exposing enzymes to elevated temperatures or organic solvents results in an irreversible loss of bioactivity due to the disruption of non-covalent interactions^43^. Thus, we selected three well-studied enzymes (HRP, (PQQ)GDH and urease, Supplementary Figs 22 and 23) and assessed whether a MOF coating could enhance their stability to extreme conditions. Accordingly, pyrogallol was added to a mixture of a ZIF-8/HRP biocomposite and trypsin in phosphate buffer at pH 7.4. Remarkably, in the presence of trypsin, the ZIF-8/HRP biocomposite essentially retained the bioactivity of HRP showing an 88% conversion of pyrogallol to purporogallin (Fig. 4). This compares with only 20% conversion for an analogous experiment where non-protected HRP was exposed to trypsin. This experiment confirms that the MOF coating acts as a protective layer for the enzyme that allows diffusion of the pyrogallol substrate (0.64 nm) through the ZIF-8 pore cavities (1.16 nm) while preventing the egress of the proteolytic agent trypsin. To benchmark the protective properties of the MOF layers, we compared them to other, commonly employed, porous protective coatings: CaCO_3_ and SiO_2_ with pores ranging from 7 to 100 nm (Supplementary Figs 24 and 25). We immersed free HRP, CaCO_3_/HRP, SiO_2_/HRP and ZIF-8/HRP biocomposites in boiling water for 1 h (Fig. 4). As expected, the free enzyme completely lost activity, while the CaCO_3_/HRP converted 39% and SiO_2_/HRP composites converted 65% (7 nm pore), 44% (20 nm pore), 17% (50 nm pore) and 13% (100 nm pore) of substrate, respectively. These values are substantially less than the 88% conversion achieved by the ZIF-8 protected HRP under the same conditions. In a further set of experiments, the same systems were immersed in boiling dimethylformamide for 1 h (Fig. 4). Once again, the free enzyme completely lost activity, while enzymes embedded in carbonate and silica particles showed 32 and 22% substrate conversion, respectively. Under these conditions, the MOF biocomposites showed a 90% conversion demonstrating again the unprecedented protective properties of the MOF layers. PXRD measurements of the biocomposites before and after the treatment in various harsh conditions showed that the peak characteristics of ZIF-8 remained unchanged (Supplementary Fig. 26), confirming the stability of the MOF biocomposites.

It has been reported that smaller pores engender higher stability of the biomacromolecuels in denaturing conditions^44^. We confirm this trend in our experiments using silica nanoparticles of varied pore sizes (Fig. 4). Therefore, the superior stability afforded by the MOF protective layer compared with CaCO_3_ and SiO_2_ can be directly related to the tight encapsulation of each biomacromolecule by the MOF structure. Indeed, each cavity within the MOF was found to range from 17% (for BSA) to 30% (for urease) larger than the size of their copying biomacromolecules as measured using SAXS (Fig. 2f, Supplementary Fig. 27). In addition, FTIR experiments of biomimetically mineralized ZIF-8 confirmed the interaction between the carbonyl groups of the protein backbone and the Zn^2+^ cations of ZIF-8 (Supplementary Fig. 28). Indeed, bonding interactions between enzymes and substrates are known to improve the robustness of biomacromolecules^45^,^46^,^47^; accordingly, these data point to a further contribution to the improved stability of the MOF-encapsulated biomolecules. Enzymatic activity studies performed on urease and (PQQ)GDH as a biomimetic mineralization agent for the growth of ZIF-8 further confirmed improved stability over the corresponding free enzymes (Supplementary Figs 22 and 23). For example, urease, which denatures above 45 °C (ref. 48), can be protected up to 80 °C exemplifying a significant relative improvement in the enzyme stabilization. Because of the size of urease (∼600 kDa)^49^ and its rapid degradation in presence of alcohols (for example, methanol)^50^, the proposed biomimetic mineralizion process overcomes the constraints of the previously reported methods that aimed to use MOFs as hosts for biomacromolecules (that is, biomolecules larger than MOF pores^27^,^28^,^29^ and/or the need for organic solvents^30^).

### Release of enzymes and proteins from ZIF-8 biocomposites

While these MOF coatings display properties advantageous for protecting enzymes in applications such as industrial catalysis and environmental remediation, the ability to control the release of bioactive macromolecules such as proteins, enzymes and DNA is highly desired, as it would provide additional opportunities in the areas of therapeutic delivery and genetic engineering^10^. Indeed, as a class, biomacromolecules offer significant advantages over small molecules such as high specificity and potency^10^. To this end, we demonstrate that the biomimetically mineralized ZIF-8 layer can be removed via simple modulation of pH^23^ (Supplementary Fig. 29), and, importantly, that the liberated biomacromolecule retains its native activity. To assess the effect of pH on the protective layer, we employed ZIF-8/FITC-BSA biocomposites. The pH-induced release profiles show that a change from 7.4 to 6 is sufficient to release the encapsulated proteins (Supplementary Fig. 30).

**Figure 5:**
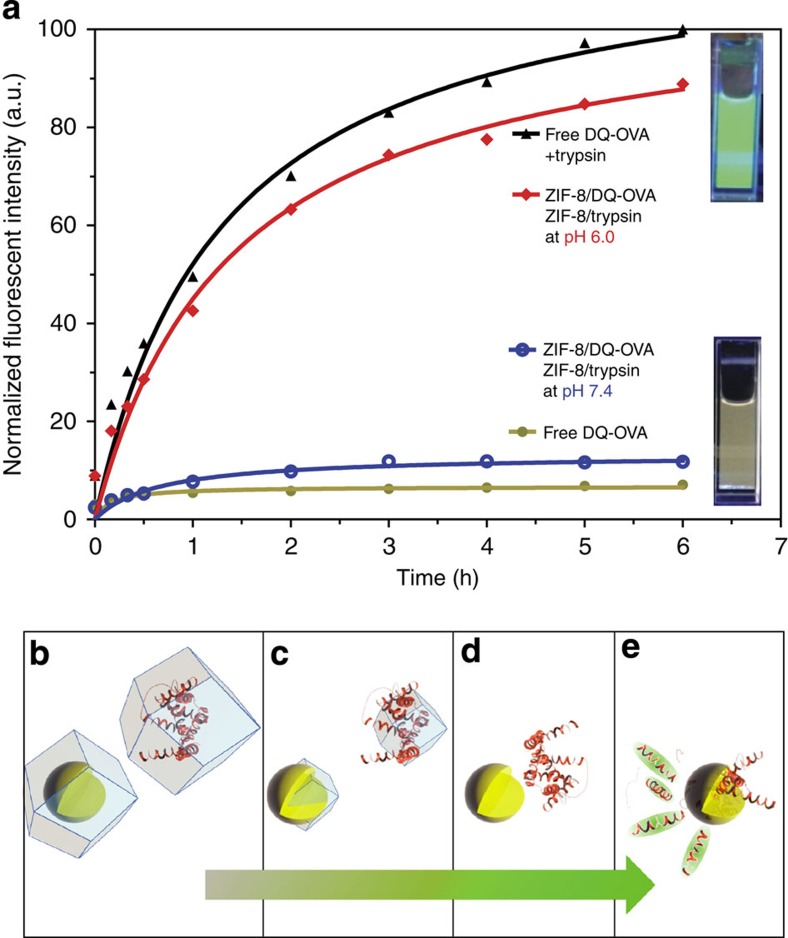
Controlled release of bioactive enzymes and proteins from ZIF-8 biocomposites. (**a**) Fluorescent measurement of the PBS solution containing biomimetically mineralized zeolitic imidazolate framework-fluorogenic protein (ZIF-8/DQ-OVA) and biomimetically mineralized zeolitic imidazolate framework-trypsin (ZIF-8/trypsin) particles. At pH 7.4, the fluorescent emission (blue line) was analogous to that of free intact DQ-OVA protein (brown line). At pH 6.0, a drastic increase in the fluorescence intensity (red line) was observed, which was attributed to the proteolysis of the DQ-OVA into luminescent fragments suggesting that trypsin and DQ-OVA have been released and are free to interact in solution. (**b**–**e**) Schematics showing the release of DQ-OVA (red) and trypsin (yellow) from ZIF-8 biocomposites at pH 6.0, and degradation of DQ-OVA into fluorescent fragments as a result of proteolysis by trypsin.

To demonstrate that the activity of the occluded biomacromolecule is maintained after the protective shell is removed, two separate biocomposites were prepared: a ZIF-8/DQ-OVA (DQ-OVA is a fluorogenic protein that shows high florescence once proteolysis occurs) and ZIF-8/trypsin (trypsin is a proteolytic enzyme). The two biocomposites were added to a PBS solution at pH 7.4. The fluorescent emission from this solution was monitored and found to be analogous to that of free intact DQ-OVA protein (Fig. 5a), thus indicating that the biomacromolecules remain stable within the MOF. When the pH of the solution was adjusted to 6.0, a drastic increase in the fluorescence intensity was observed which is attributed to the proteolysis of the DQ-OVA into luminescent fragments; suggesting that trypsin and DQ-OVA have been released from their protective MOF coatings and are free to interact in solution (Fig. 5b–e). This result confirms that the bioactivity of both the trypsin and the DQ-OVA is preserved following their release from ZIF-8 and, along with the combined low toxicity of the ZIF-8 coating^23^,^33^, supports the potential application of MOFs in the area of biobanking or drug delivery.

## Discussion

Here we have demonstrated the first example of biomimetic mineralization of MOFs. Furthermore, we show the presence of a crucial synergistic interaction in the solution between the MOF precursors and the biomacromolecules. This involves the biomacromolecule concentrating the MOF building blocks, which leads to nucleation of porous crystals. In this process, the biomacromolecules regulate the crystal size, morphology and crystallinity while encapsulating itself within the porous crystal and concomitantly generating new cavities that both tightly surround the biomacromolecules and form bonding interactions with the protein backbone. This mechanism is shown to protect a variety of biomacromolecules (for example, proteins and enzymes) from inhospitable environments. Enzymes encapsulated in MOFs were shown to maintain their activity even after the exposure to extreme conditions. This protective capacity is remarkable, and far exceeds that of current materials used for this purpose such as CaCO_3_ and mesoporous silica. The controlled release of the biomacromolecules as a bioactive cargo from its protective MOF coating can be achieved via simple pH modification. We anticipate that this bioinspired approach for protecting and delivering functional biomacromolecules will facilitate their application in areas where stability has previously been an issue, such as industrial biocatalysis, biopharmaceuticals and biobanking.

## Methods

### Materials

Cy3-labelled Oligonucleotide (50 bases, MW: 16 kDa) was purchased from Trilink Biotechnologies Inc. (San Diego, California, USA). DQ-ovalbumin (DQ-OVA) was obtained from Life Technologies (VIC, Australia). All other reactants were purchased from Sigma-Aldrich and used without further modification.

### Biomimetic mineralization of ZIF-8/proteins

Various amounts of the appropriate proteins (e.g. 10, 50, 100, 200 mg BSA) and enzymes (e.g. 80 mg HRP) were added into a solution of 2-methylimidazole (160 mM, 20 ml, pH 10.3) in deionized water. (For insulin, 10 mg insulin was added in water, the pH was adjusted to 3–4 with HCl (20 mM) to completely dissolve the insulin and adjusted back to pH 10.3 before the addition of 2-methylimidazole (160 mM)). A separate solution of zinc acetate dihydrate dissolved in deionised water (40 mM, 20 ml) was also prepared. These two solutions were combined and then agitated for 10 s. The resulting solution was aged for 12 h at room temperature. The obtained precipitate was recovered by centrifugation at 6,000 r.p.m. for 10 min and then washed, sonicated, and centrifuged twice each in water followed by ethanol. The encapsulation efficiency of proteins in ZIF-8 was determined by fluorescent spectrophotometry using a pre-determined calibration curve of FITC-labelled proteins. Protein encapsulation efficiency: BSA ∼100%, HSA ∼100%, ∼100%, lysozyme ∼96%, HRP ∼100%, ribonuclease A ∼86%, haemoglobin ∼90%, trypsin ∼96%, lipase ∼88%, insulin ∼86%, PQQ-GDH ∼82%, urease ∼95%.

### Biomimetic mineralization of ZIF-8/DNA

Cy3-labelled oligonucleotide (200 μl; 20.8 μM) was added into a solution of 2-methylimidazole (160 mM, 0.5 ml) in deionized water. A separate solution of zinc acetate dissolved in deionised water (40 mM, 0.5 ml) was prepared. These two solutions were then mixed and vortexed for 10 s. The mixture was aged for 12 h at room temperature. The obtained precipitate was recovered by centrifugation at 6,000 r.p.m. for 10 min and then washed, sonicated, and centrifuged twice each in water followed by ethanol. The encapsulation efficiency (75%) of the DNA in ZIF-8 was determined using a fluorescence spectrophotometer collecting the emission at 561 nm (Cy3 emission maximum) from a pre-determined calibration curve, by measuring the concentrations of the DNA in the precursor solution and in the supernatant of the obtained crystals.

### Biomimetic mineralization of HKUST-1

Benzene-1,3,5-tricarboxylic acid (BTC) was dissolved in ethanol (53.45 mM, 20 ml). A separate solution of copper(II) nitrate dissolved in deionized water (40.09 mM, 20 ml) was also prepared. These two solutions were then mixed and vortexed for 10 s. BSA solution (1.2 ml; 10 mg ml^−1^ in MQ) was then added into the mixture. The mixture was aged for 12 h at room temperature. The suspension was centrifuged at 6,000 r.p.m. for 20 min, and subjected to centrifugation–wash cycles three times using ethanol.

### Biomimetic mineralization of Eu-BDC and Tb-BDC

Disodium terephthalate salt was prepared following the procedure from the literature^51^. 5 g of terephthalic acid (BDC) was dissolved in deionized water to which 2.32 g of sodium hydroxide was added. The resulting solution was evaporated to dryness and the solid was then resuspended in ethanol and refluxed for 1 h before filtering, washing with water and drying. The disodium salt of terephthalic acid was then dissolved in deionized water (10 mM, 20 ml) to which the 20 mg of BSA was dissolved. The EuCl_3_.6H_2_O or TbCl_3_.6H_2_O was also dissolved in deionized water (10 mM, 20 ml). The lanthanide salt solution and the BSA ligand solution were then mixed and vortexed for 10 s. The solution was gently agitated for 12 h before recovering the precipitate by centrifugation at 6,000 r.p.m. for 20 min and then washing and centrifuging in ethanol three times.

### Biomimetic mineralization of MIL-88A

Various amount of BSA (2, 4, 8, 16 mg ml) was dissolved in fumaric acid solution in deionized water (25 mM). A separate solution of FeCl_3_·6H_2_O (25 mM) was prepared and immediately mixed with equal volumes of BSA-containing fumaric acid solution and vortexed for 10 s. The solution mixture was aged for 7 days at room temperature. The suspension was centrifuged at 6,000 r.p.m. for 20 min, and subjected to centrifugation–wash cycles three times using ethanol.

### Bioactivity of ZIF-8/HRP

The obtained crystals were firstly redispersed in SDS (0.2 g in 2 ml deionized water) solution at 70 °C for 10 min to wash off the free enzymes on the crystal surface^52^,^53^, followed by three centrifugation/wash cycles in water. The activity of HRP (1.11.1.7; lot no. 125F-9640, HRPO Type U; Sigma Chemical Co., St. Louis, MO) was determined by measuring the rate of decomposition of hydrogen peroxide with pyrogallol as the hydrogen donor, which can be converted to a yellowish product, purpurogallin^54^. In a typical assay, solution A containing 76 μl KH_2_PO_3_ (100 mM, pH 6.0), 38 μl H_2_O_2_ (5% w/w in deionized water), 76 μl pyrogallol (5% w/w in deionized water) and 1.8 ml of PBS buffer (pH 7.4) was prepared. To solution A, ZIF-8/HRP crystals were added, and the absorbance of the solution was immediately monitored at 420 nm using a UV-Vis spectrophotometer at 30 s increments. In an enzymatic activity assay using free HRP, the amount of free enzymes introduced into solution A was adjusted to be equal to the amount of enzymes loaded into ZIF-8/HRP, as determined from the loading efficiency.

### Bioactivity of ZIF-8/(PQQ)GDH

The obtained crystals were redispersed in a SDS (10%w/w in deionised water, 2 ml) solution at 70 °C for 10 min to wash off the free enzymes on the crystal surface,^52^,^53^ followed by three centrifugation/wash cycles in water. The activity of (PQQ)GDH was determined using phenazine methosulfate as an electron acceptor^55^,^56^. In a typical assay, solution B containing 1 ml glucose (20 mM in 10 mM MOPS buffer pH 7.0) 10 μl 2,6-dichloroindophenol (0.1 mM in deionized water), 10 μl phenazine methosulfate (0.06 mM in deionised water) was prepared. To solution B, ZIF-8/(PQQ)GDH crystals were added, and the absorbance of the solution was immediately monitored at 600 nm using a UV-Vis spectrophotometer at 30 s increments. In an enzymatic activity assay using free (PQQ)GDH, the amount of free enzymes introduced into solution B was adjusted to be equal to the amount of enzymes loaded into ZIF-8/(PQQ)GDH, as determined from the loading efficiency.

### Bioactivity of ZIF-8/urease

The activity of ZIF-8/urease was determined by measuring the pH increase as a result of urea conversion to ammonia, using phenol red as a pH indicator^57^. Phenol red solution was prepared by dissolving 10 mg phenol red in 284 μl of NaOH solution (0.1 M), and made up to a final volume of 10 ml with deionized water. In a typical assay, 10 μl phenol red solution, 990 μl urea solution (0.5 M) and ZIF-8/urease was added into an ultraviolet–visible cuvette, and the absorbance of the solution was monitored at 560 nm using a UV-Vis spectrophotometer at 30 s increments.

### pH-triggered release of biomacromolecules

ZIF-8/FITC-BSA (1 mg) was dispersed in 2 ml of pH-adjusted PBS at pH 7.4 or pH 6.0 at 37 °C under gentle agitation. Over 24 h, at regular time intervals, the crystal dispersion was centrifuged at 6,000 r.p.m. for 20 min, and the fluorescence intensity of the released FITC-BSA was assessed by monitoring the fluorescent intensity from the supernatant using a fluorescence spectrophotometer.

### Bioactivity from released enzymes

Equal volumes of colloidal solution containing ZIF-8/trypsin and ZIF-8/DQ-OVA were added to 2 ml pH-adjusted PBS at pH 7.4 or pH 6.0 at 37 °C under gentle agitation. The fluorescent emission at 515 nm from the BODIPY dye in the solution that resulted from the proteolysis of DQ-OVA by trypsin was constantly monitored using a fluorescence spectrophotometer.

## Additional information

**How to cite this article**: Liang, K. *et al*. Biomimetic mineralization of metal-organic frameworks as protective coatings for biomacromolecules. *Nat. Commun.* 6:7240 doi: 10.1038/ncomms8240 (2015).

## Supplementary Material

Supplementary Information Supplementary Figures 1-30, Supplementary Methods and Supplementary References

## Figures and Tables

**Figure 1:**
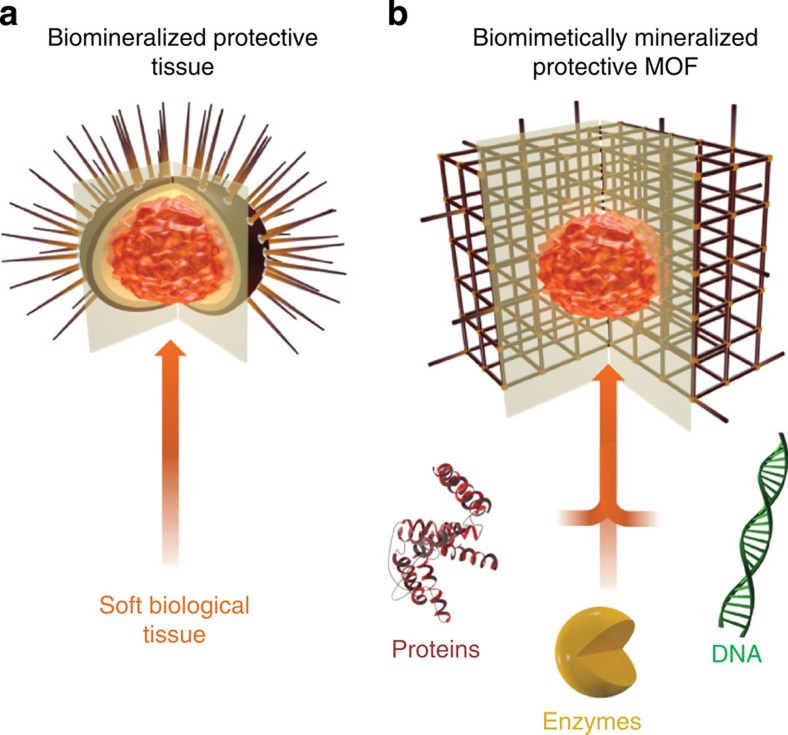
Schematic illustration of biomimetically mineralized MOF. (**a**) Schematic of a sea urchin; a hard porous protective shell that is biomineralized by soft biological tissue (**b**) Schematic of a MOF biocomposite showing a biomacromolecule (for example, protein, enzyme or DNA), encapsulated within the porous, crystalline shell.
