# Supplementary material for: Biomimetic mineralization of metal-organic frameworks as protective coatings for biomacromolecules
Source: Nat Commun. 2015 Jun 4;6:7240. doi: 10.1038/ncomms8240 (PMC4468859; doi:10.1038/ncomms8240)
Supplement: Supplementary Information — Supplementary Figures 1-30, Supplementary Methods and Supplementary References [file ncomms8240-s1.pdf]

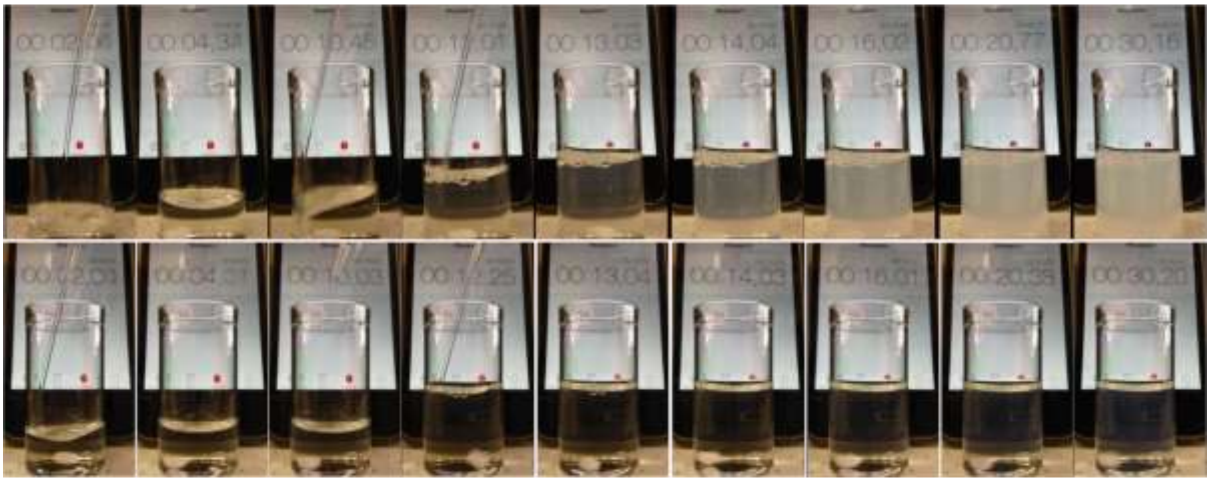

**Supplementary Figure 1.** Sequential video frames depicting the rapid MOF formation by biomimetic mineralization approach. **Top row**, reaction conducted by adding BSA to the ZIF-8 MOF precursor in water. **Bottom row**: solution containing the MOF precursors without BSA. The first 2 frames (2 and 4 seconds shows the addition of water containing the $Zn^{2+}$ cations into an empty vessel (zinc acetate dissolved in deionised water (40 mM, 20 mL)). The next 2 frames (10 and 12 seconds) show the addition of the water containing HmIm (2-methylimidazole (160 mM, 20 mL)). In presence of BSA (first row, 1 mg) the solution becomes less transparent almost instantaneously (1 s) and then the opacity increases up to 30 s reaction. Without BSA (bottom row) no changes in the transparency are detected because the MOF does not form during the investigated reaction time.

Confocal Scanning Laser Microscopy (CLSM) was used to investigate the presence of luminescent-tagged proteins within the ZIF-8 MOF crystals.

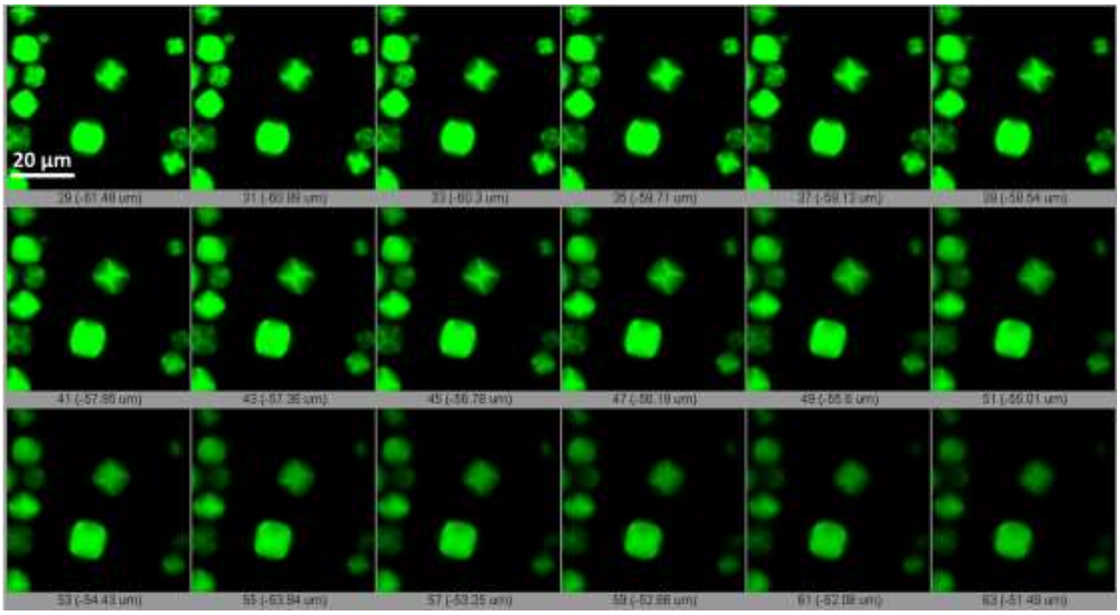


**Supplementary Figure 2.** CLSM images of a ZIF-8/BSA particle at a series of focal planes measured every 126 nm along the z-axis. This study showed the even distribution of FITC-labelled BSA within ZIF-8 crystals.

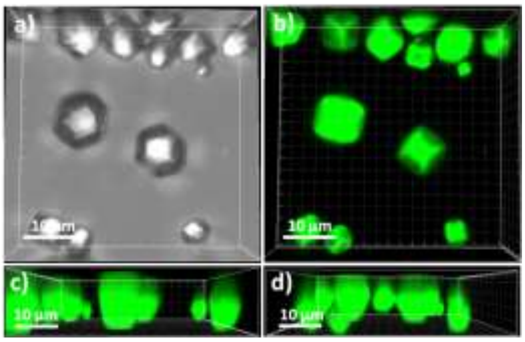


**Supplementary Figure 3.** a) Differential interference contrast (DIC) image of top view of ZIF-8/BSA crystals. b) CLSM image of 3D top view of ZIF-8/BSA. c) and d) CLSM image of 3D lateral view of ZIF-8/BSA particles. These images have been obtained by overlaying the different sections collected during the confocal scanning along the z axis.

A confocal investigation was performed on a pure ZIF-8 post-exposed to FITC-BSA, to study the diffusion abilities of the proteins within the MOFs.

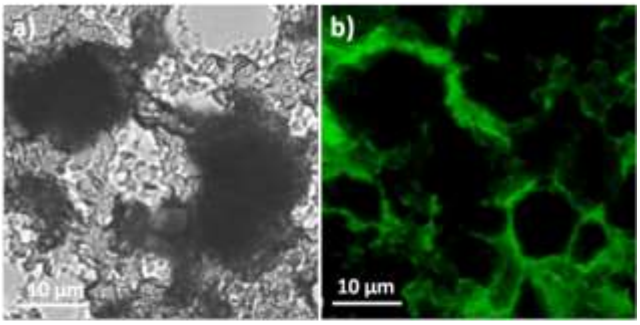


**Supplementary Figure 4.** DIC (a) and CLSM (b) images of ZIF-8 crystals after post-exposure in FITC-BSA solution. The fluorescence was only observed on the surface of the particles (the focal plane in the images above shows a cross section of the crystals), suggesting that the BSA was not able to diffuse into the ZIF-8 crystals.

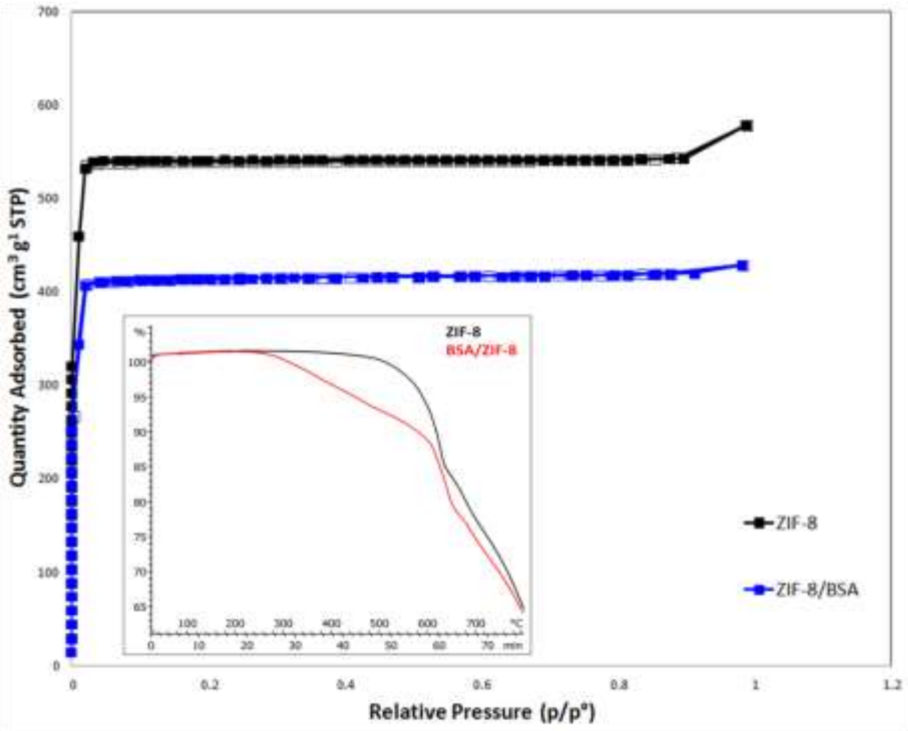


**Supplementary Figure 5.** $N_2$ adsorption/desorption curves at 77 K for biomimetically mineralized ZIF-8/BSA and standard ZIF-8, giving BET surface areas of 1381 and 1776 $m^2 g^{-1}$, respectively. Closed symbols denote the adsorption branch of the isotherm and open symbols the desorption branch. Inset: TGA analysis of BSA-biomimetically mineralized ZIF-8 and standard ZIF-8.

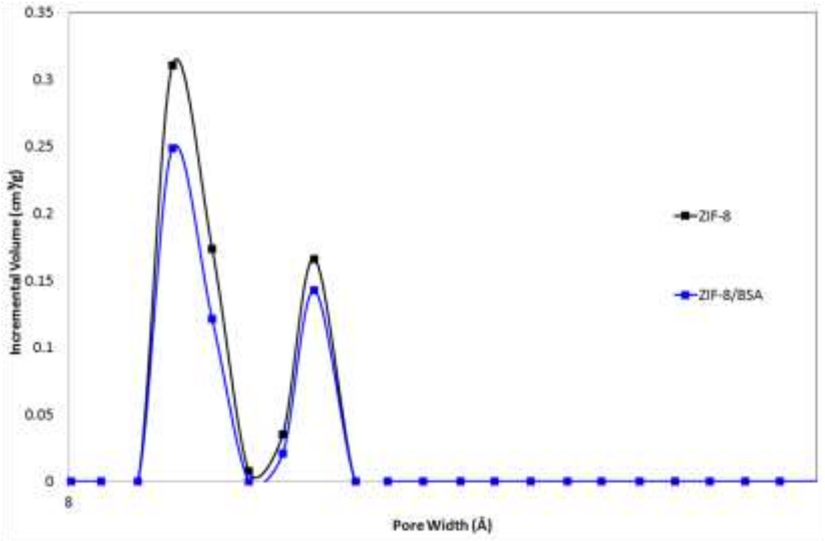


**Supplementary Figure 6.** DFT pore size distribution of biomimetically mineralized ZIF-8/BSA and standard ZIF-8. There is no apparent shift in the pore size which suggests that the ZIF-8 forms around the biomolecules.

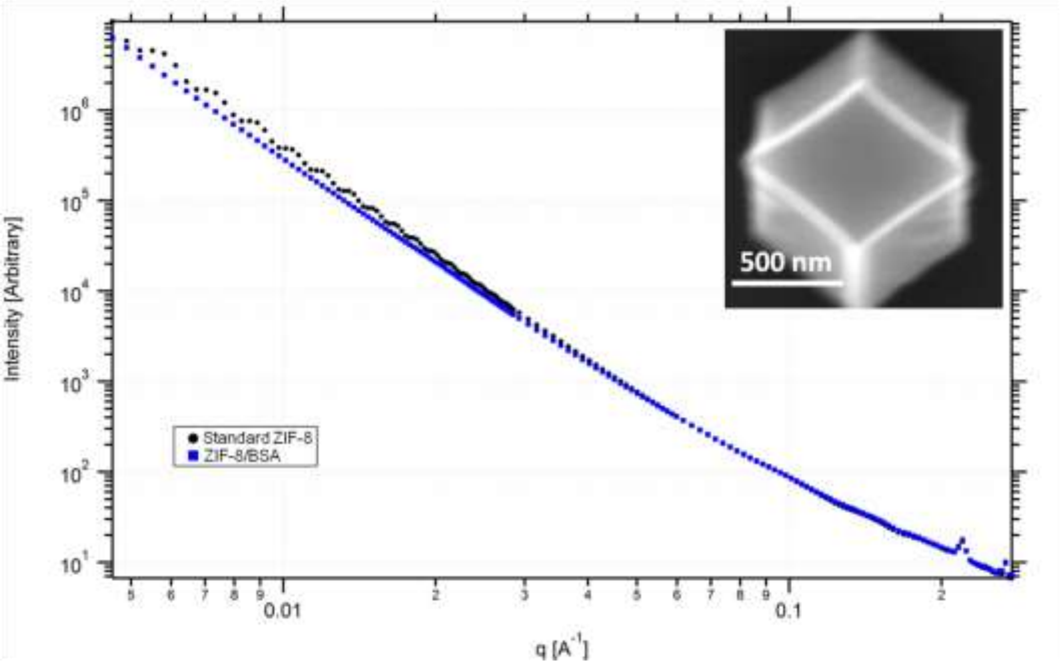


**Supplementary Figure 7.** SAXS plot of the intensity (counts) versus q(Å$^{-1}$). Data are collected for standard ZIF-8 and biomimetically mineralized ZIF-8/BSA biocomposites.

The oscillations in the scattering pattern for the standard ZIF-8 indicate the presence of discrete dispersed particles with a narrow particle size distribution, consistent with parallel-piped shaped particles with edges 400 nm long (inset SEM image). Samples synthesised in the presence of proteins show only power law slopes, indicating that the particles are aggregated and polydisperse in size.

At high protein concentrations (20 mg), a more evident Guinier knee is observed and can be fitted using the Unified model (Beaucage[1]) with a radius of gyration (Rg)[2] of 35 (± 5) Å, which is slightly larger than that of BSA (29.9 Å).[3] This result is consistent with pores formed within the MOF structure due to incorporation of largely monomeric BSA (**Figure 2f** in the main text).

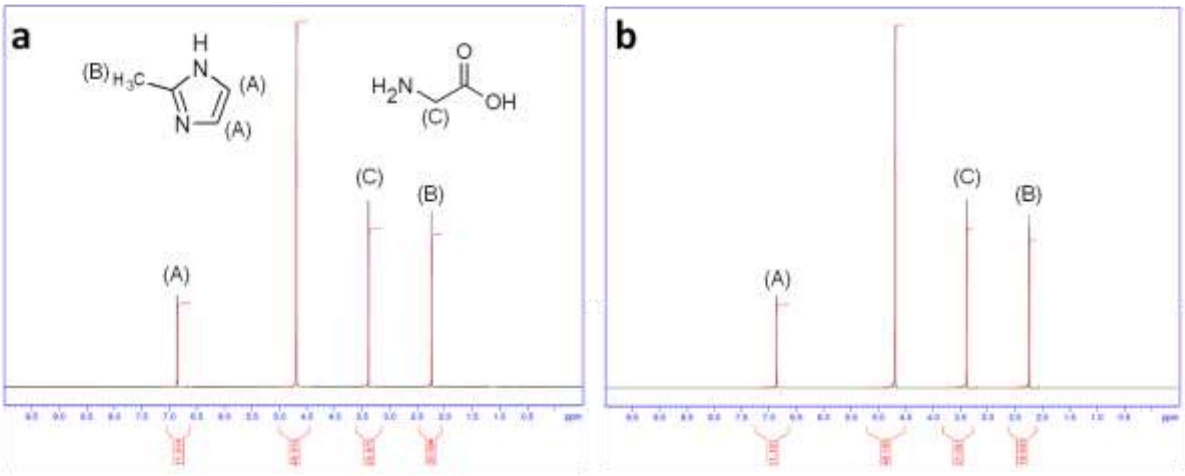


**Supplementary Figure 8**. $^{1}$H NMR spectra in $D_2O$ of the supernatant containing HmIm using glycine as an internal standard a) before and b) after the incubation of BSA. In order to separate the BSA from HmIm solution for NMR measurements before and after the incubation with the ligand, BSA was immobilized on mesoporous silica particles. The amount of HmIm adsorbed to BSA was determined by calculating the difference of HmIm integral peaks before and after the incubation.

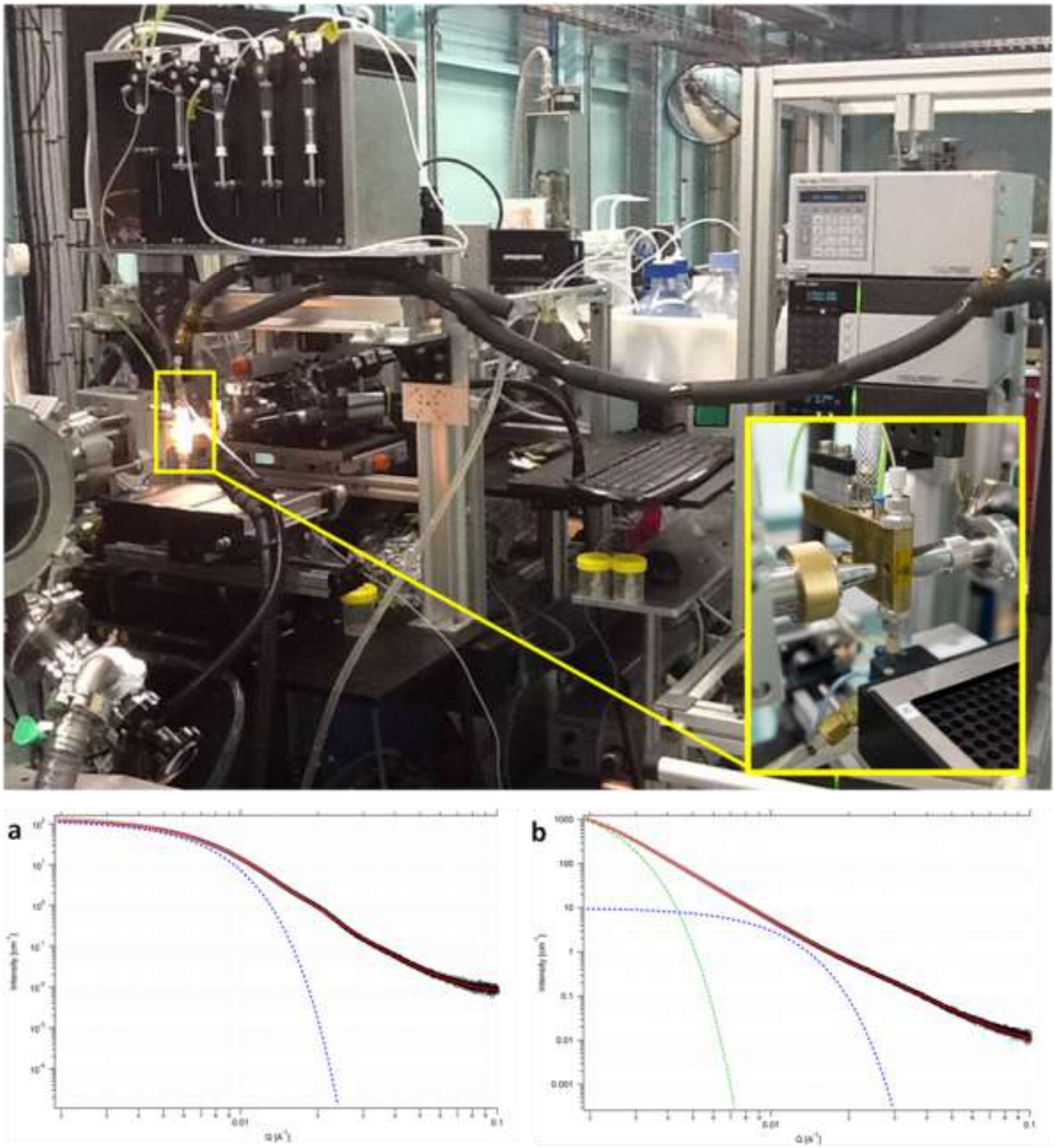


**Supplementary Figure 9**. In situ experiment of biomimetic mineralization of ZIF-8 (ZIF-8/BSA) performed at the Australian Synchrotron (SAXS beamline). The set-up used peristaltic pumps to perform the reaction in a capillary (reaction chamber) with the different reactants to perform the reaction under X-ray irradiation. The inset of the photo shows a detail of the capillary placed between the incident beam and the path through the detector (Pilatus 1M). SAXS plot of the intensity (counts) versus q ($Å^{-1}$) at 30 s after mixing the aqueous ZIF-8 precursor solution. (a) without the presence of BSA, (b) in the presence of

BSA (1mg/mL). Unified fit (red) of experimental data (black), Guinier component of unified fit showing the presence of small particles (blue, Rg = 30 Å) and the new generation of larger particles (green, Rg = 100 Å). The experiment was performed in a pulsed pumping mode to increase the statistics over a large volume of solution.

Upon mixing the aqueous MOF precursor solutions (2-methylimidizole and zinc acetate), a monomodal distribution of small particles (Rg = 30 Å) was formed within 180 s. After this reaction time, no further evolution of the particle size distribution was detected. In contrast, when BSA (1 mg/ml) was used as biomimetic mineralization agent, a second generation of bigger particles (Rg = 100 Å) was rapidly formed. Within 30 s no further changes on the particle distribution was detected. This information confirms the role of the BSA as biomimetic mineralization agent.

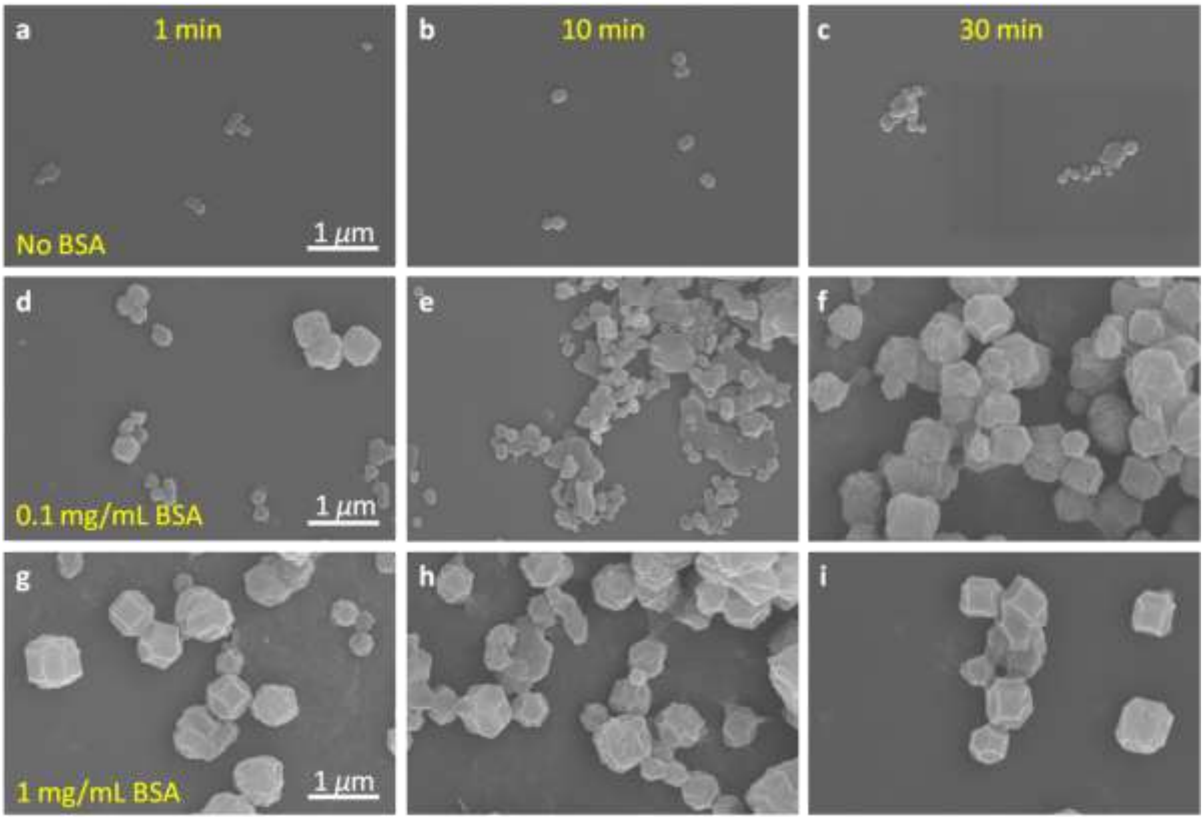


**Supplementary Figure 10.** SEM images of time evolution of the ZIF-8 crystal growth, (a-c) without BSA, (d-f) in the presence of 0.1 mg/mL BSA, and (g-i) in the presence of 1 mg/mL BSA. Without the presence of BSA, only a small amount of particles (~50-100 nm) were detected under SEM. However, in the presence of BSA, larger particles (~1 μm) were observed within 30 s.

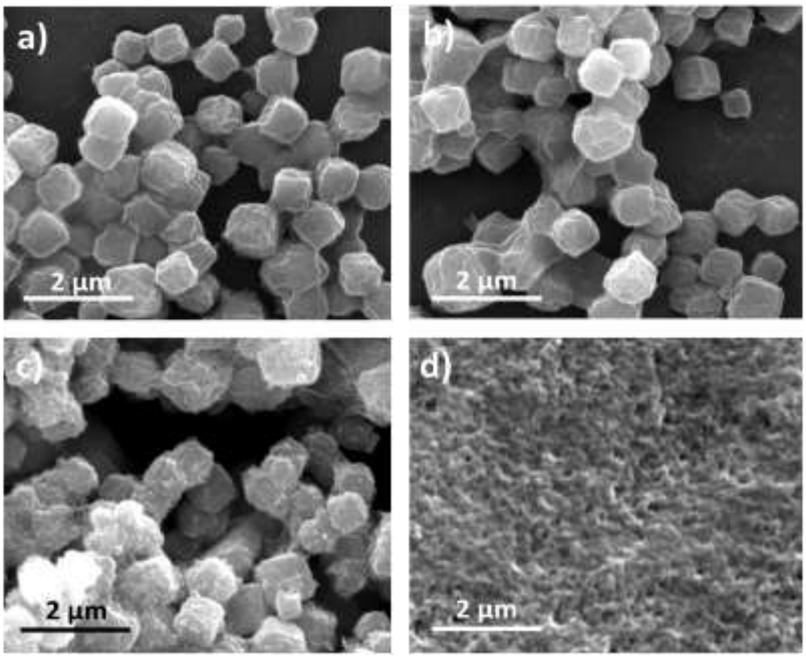


**Supplementary Figure 11.** SEM images of biomimetically mineralized ZIF-8 using various amounts of BSA. 1 mg (a), 5 mg (b), 10 mg (c), and 20 mg (d) BSA was dissolved in aqueous solutions of HmIm (160 mM, 2 mL) before mixing with aqueous solutions of zinc acetate (40 mM, 2 mL) at room temperature.

| BSA [mg] | EDS wt% C | EDS wt% Zn | wt% C/Zn |
|---|---|---|---|
| 0 | 46 | 28.9 | 1.59 |
| 1 | 39.3 | 23.4 | 1.68 |
| 5 | 42.9 | 19.6 | 2.19 |
| 10 | 42.5 | 14 | 3.04 |
| 20 | 33.8 | 6.5 | 5.2 |

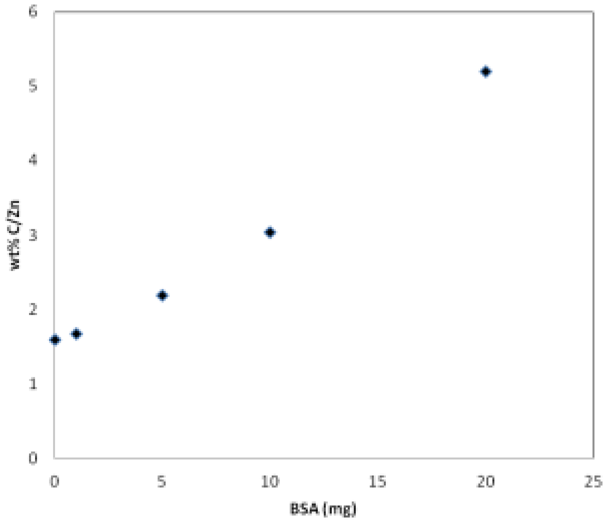


**Supplementary Figure 12.** EDS measurement of biomimetically mineralized ZIF-8 using various amounts of BSA. 1 mg (a), 5 mg (b), 10 mg (c), and 20 mg (d) BSA was dissolved in aqueous solutions of HmIm (160 mM, 2 mL) before mixing with aqueous solutions of zinc acetate (40 mM, 2 mL) at room temperature. This result showed the wt% ratio of C/Zn increases according with the increase of the BSA amount encapsulated during the biomimetic mineralization of ZIF-8.

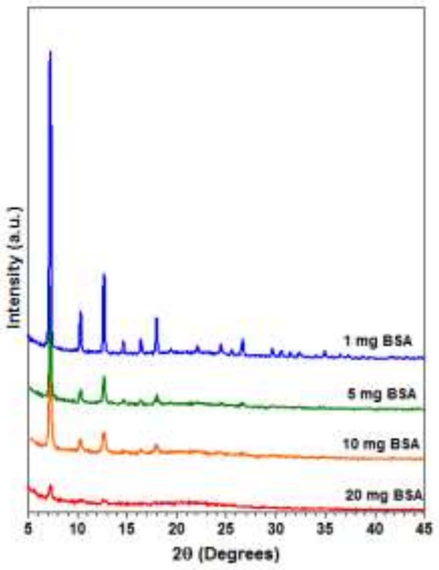


**Supplementary Figure 13.** XRD pattern showing the decrease of crystallinity with increasing concentration of BSA used in the MOF biomimetic mineralization. 1 mg (blue), 5 mg (green), 10 mg (orange), and 20 mg (red) BSA was dissolved in aqueous solutions of HmIm (160 mM, 2 mL) before mixing with aqueous solutions of zinc acetate (40 mM, 2 mL) at room temperature.

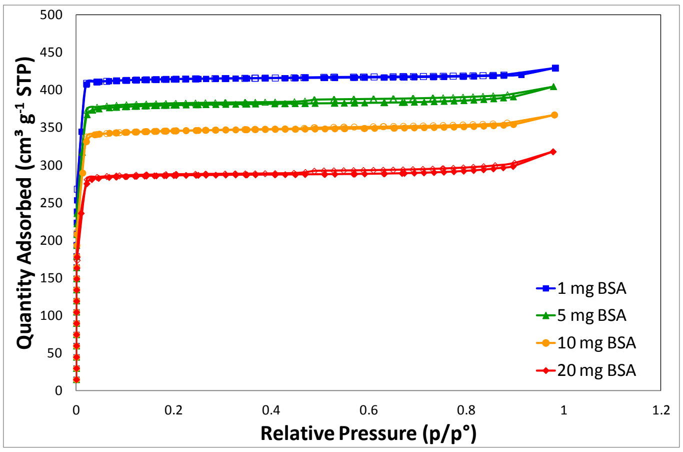


**Supplementary Figure 14.** $N_2$ adsorption/desorption curves at 77 K for biomimetically mineralized ZIF-8 using, 1 mg, 5 mg, 10 mg, and 20 mg of BSA, giving surface areas of 1381, 1268, 1154, and 956 $m^2$ $g^{-1}$, respectively. Closed symbols denote the adsorption branch of the isotherm and open symbols the desorption branch.

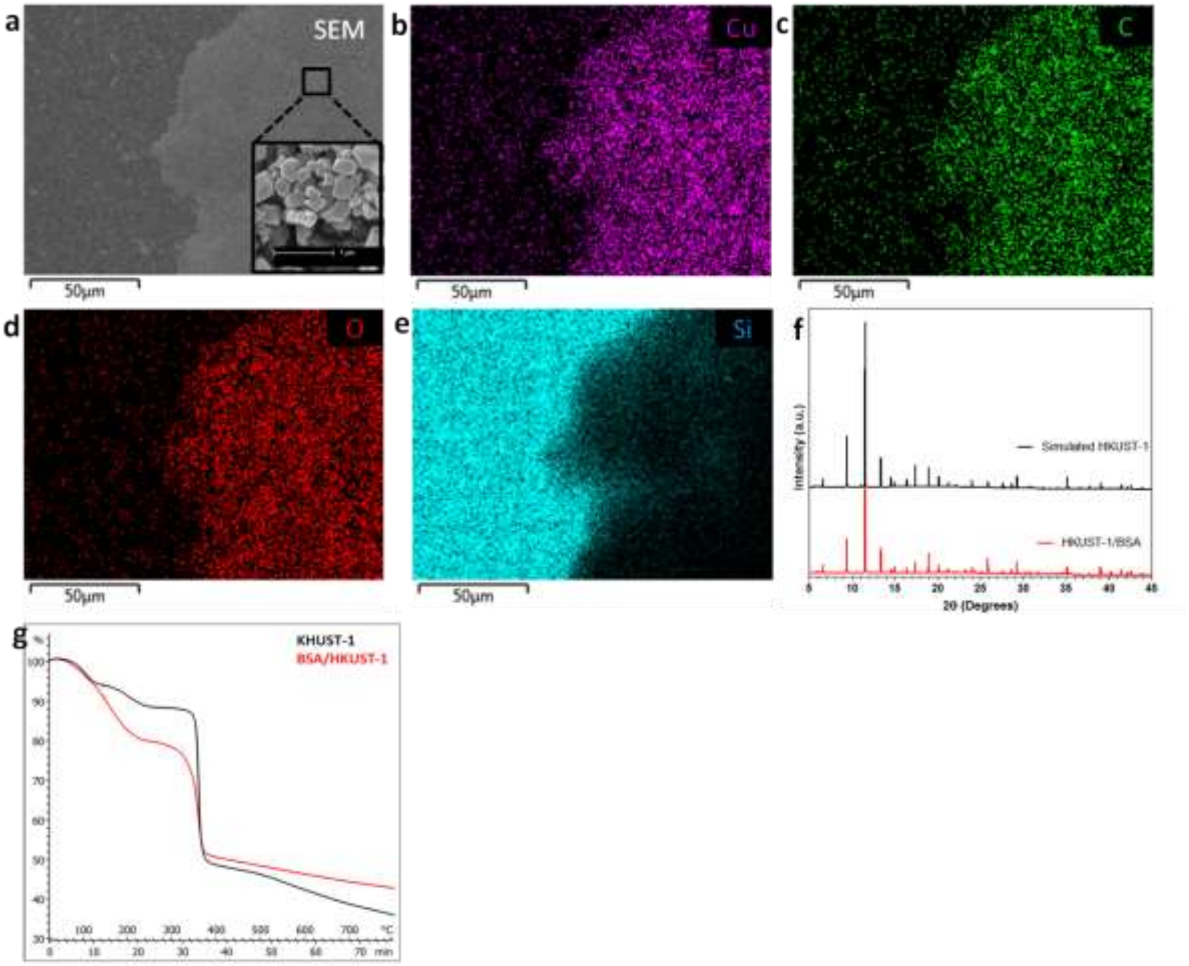


**Supplementary Figure 15.** a) SEM and b-e) EDX images of BSA-biomimetically mineralized HKUST-1 crystals. f) Synchrotron XRD measurement of BSA-biomimetically mineralized HKUST-1 crystals and simulated HKUST-1 pattern. The formation of the crystals in the presence of BSA was detected within the first minute. In contrast, in the absence of BSA, no crystal formation was observed. g) TGA analysis of BSA-biomimetically mineralized HKUST-1 and standard HKUST-1.

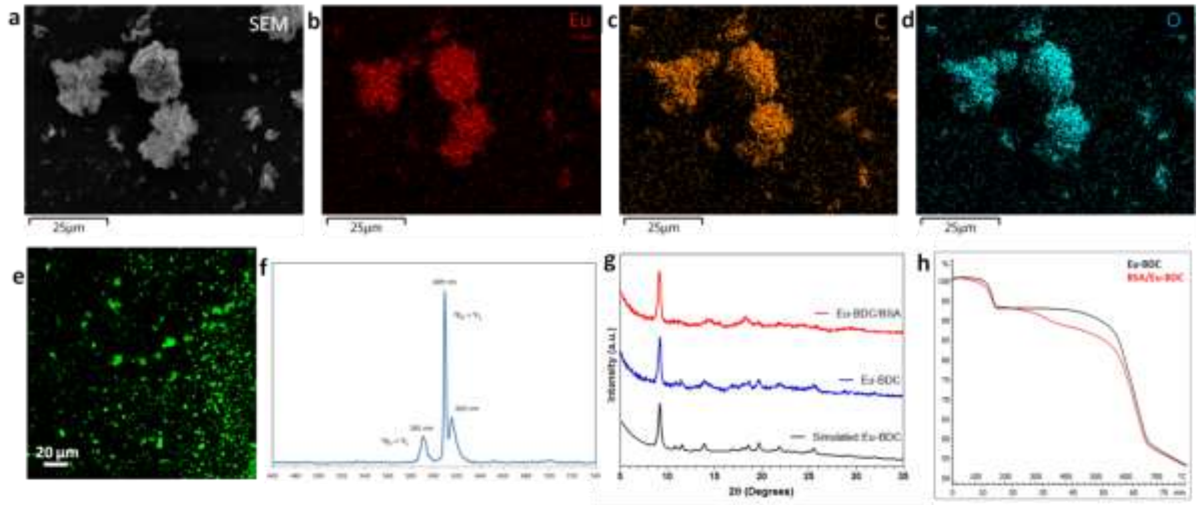


**Supplementary Figure 16.** a) SEM and b-d) EDX images of BSA-biomimetically mineralized Eu-BDC crystals. e) CLSM image showing the FITC-labelled BSA within Eu-BDC crystals. f) Fluorescent emission spectra of Eu-BDC/BSA. g) XRD measurements of BSA-induced Eu-BDC crystals and Eu-BDC synthesised using the literature reported method,[4] and Eu-BDC pattern simulated via the Pawley method[5] using the lattice parameters reported by Daiguebonne.[4] The formation of the crystals in the presence of BSA was detected within 5 min. In contrast, in the absence of BSA, no crystal formation was observed at this time frame. h) TGA analysis of Eu-BDC/BSA and standard Eu-BDC.

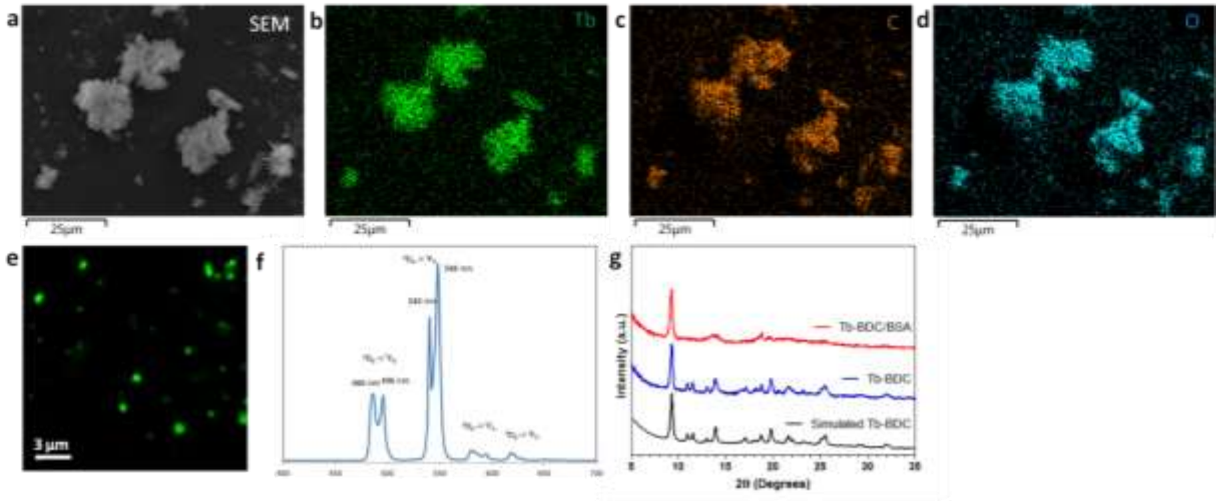


**Supplementary Figure 17.** a) SEM and b-d) EDX images of BSA- biomimetically mineralized Tb-BDC crystals. e) CLSM image showing the FITC-labelled BSA within Tb-BDC crystals. f) Fluorescent emission spectra of Tb-BDC/BSA. g) XRD measurements of BSA-biomimetically mineralized Tb-BDC crystals and Tb-BDC synthesised using the reported literature method,[4] and Tb-BDC pattern simulated via the Pawley method[5] using the lattice parameters reported by Daiguebonne.[4] The formation of the crystals in the presence of BSA was detected within 5 min. In contrast, in the absence of BSA, no crystal formation was observed at this time frame.

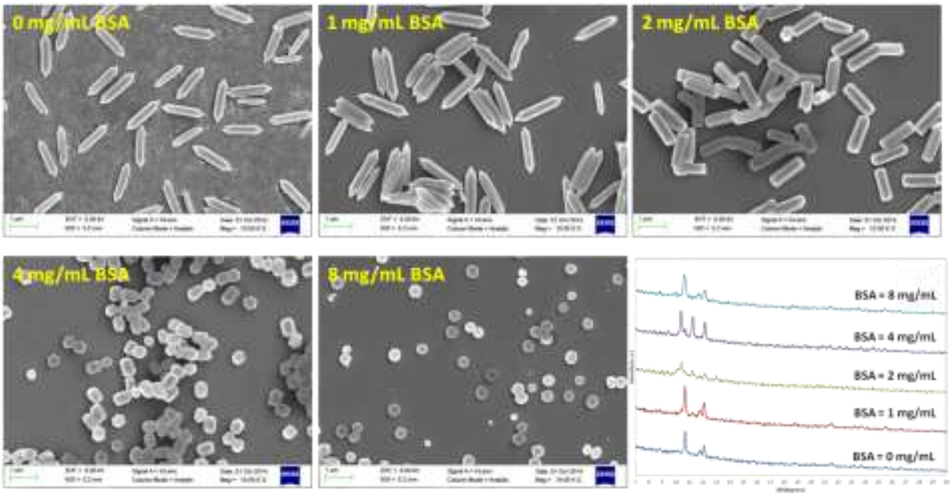


**Supplementary Figure 18.** SEM and PXRD measurements of BSA-biomimetically mineralized MIL-88A using 0, 1, 2, 4, and 8 mg/mL BSA, respectively.

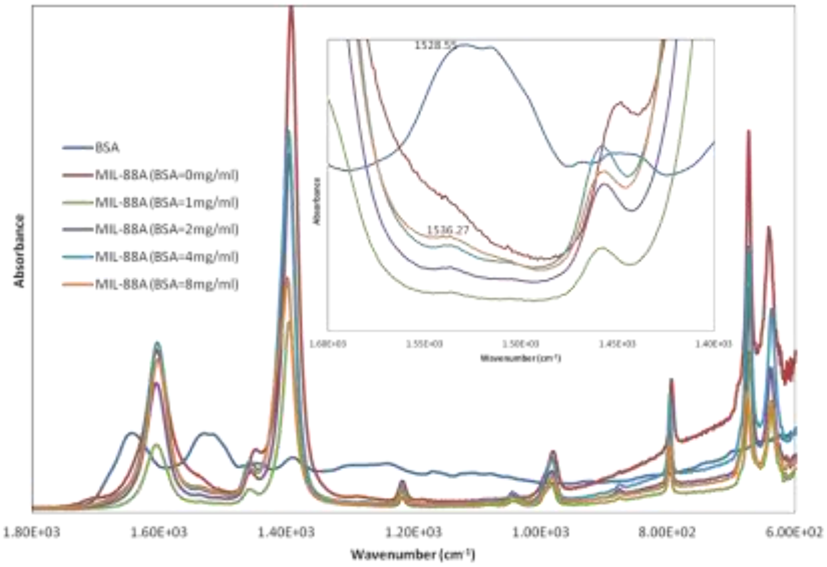


**Supplementary Figure 19**. FTIR spectra of BSA-biomimetically mineralized MIL-88A crystals using 0, 1, 2, 4, and 8 mg/mL BSA, respectively. The presence of the peak near 1528 $cm^{-1}$ (N-H bending vibration/C-N stretching vibration) which is characteristic of BSA is present in all the biomimetically mineralized MIL-88A samples, confirming the encapsulation of BSA during the biomimetic mineralization process. Moreover, there is a slight shift of 1528 $cm^{-1}$ peak toward higher wavenumbers (1536 $cm^{-1}$) in the biomimetically mineralized MIL-88A samples, indicating the interaction of Fe cations with the BSA protein.

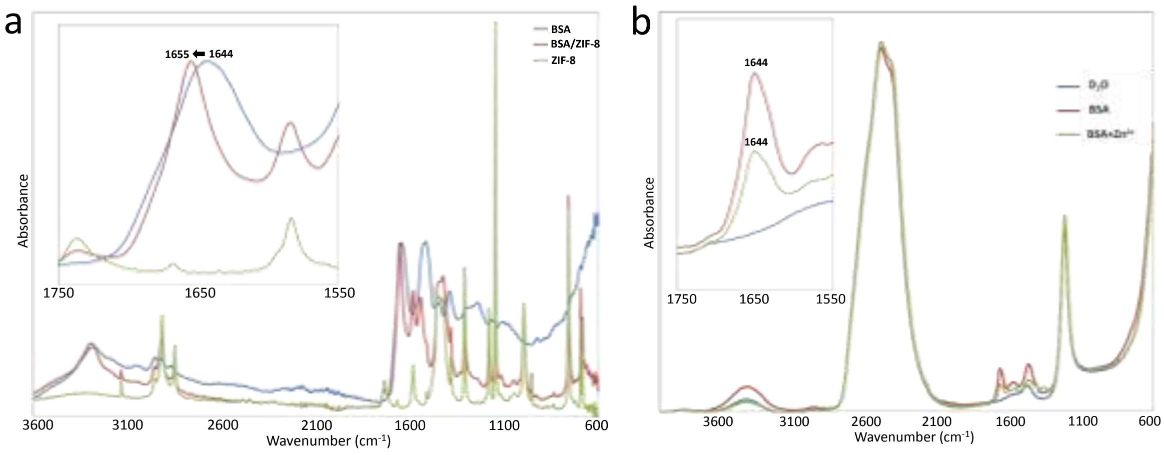


**Supplementary Figure 20.** FTIR spectra of (a) BSA (blue), BSA-biomimetically mineralized ZIF-8 (red), and standard ZIF-8 (green); (b) BSA in $D_2O$ (red), BSA and $Zn^{2+}$ in $D_2O$ (green), and $D_2O$ (blue). Insets show the zoomed in spectra.

The presence of the peak around 1640 $cm^{-1}$ for BSA/ZIF-8 (amide I vibration, C-O stretch) corresponding to α-helix[6,7] of the proteins are observed in all the biomimetically mineralized ZIF-8 samples, confirming the incorporation of the proteins during the biomimetic mineralization process. There is a slight shift of the amide I peak towards higher wavenumbers in the biomimetically mineralized ZIF-8 samples. In contrast, BSA incubated with only $Zn^{2+}$ ions showed no obvious shift of the amide I peak. The shift of amide I peak in biomimetically mineralized ZIF-8 is evidence of a direct protein-MOF interaction due to the coordination between $Zn^{2+}$ and the carbonyl groups on the proteins.[8–10]

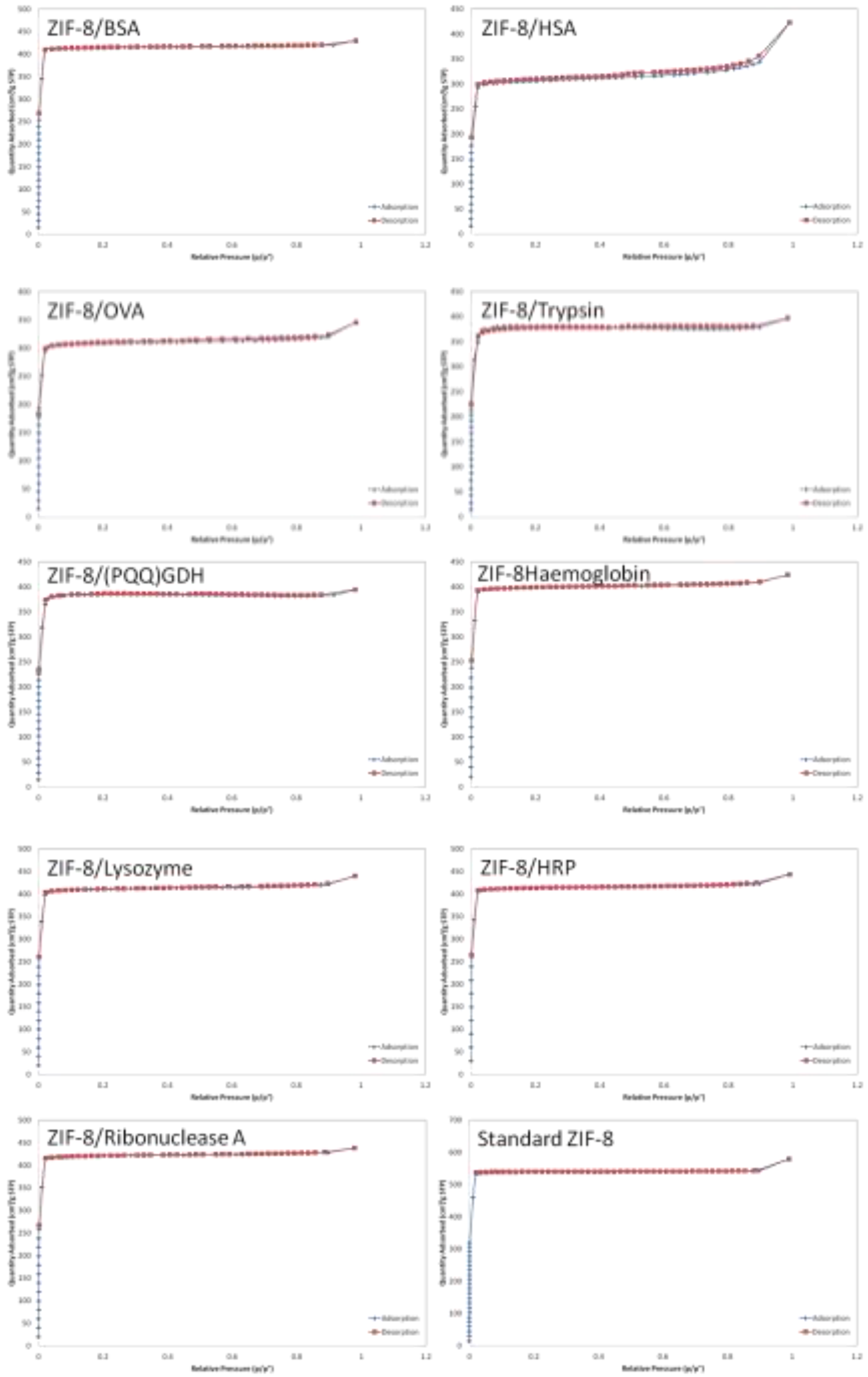


**Supplementary Figure 21**. $N_2$ adsorption/desorption curves at 77 K for biomimetically mineralized ZIF-8 using 1 mg a) BSA, b) HSA, c) OVA, d) trypsin, e) (PQQ)GDH, f) haemoglobin, g) lysozyme, h) HRP, i) ribonuclease A, giving surface areas of 1381, 1025, 1031, 1307, 1278, 1329, 1370, 1376, and 1404 $m^2\ g^{-1}$, respectively. j) ZIF-8 crystals synthesized using standard protocols in methanol with a surface area of 1776 $m^2\ g^{-1}$.

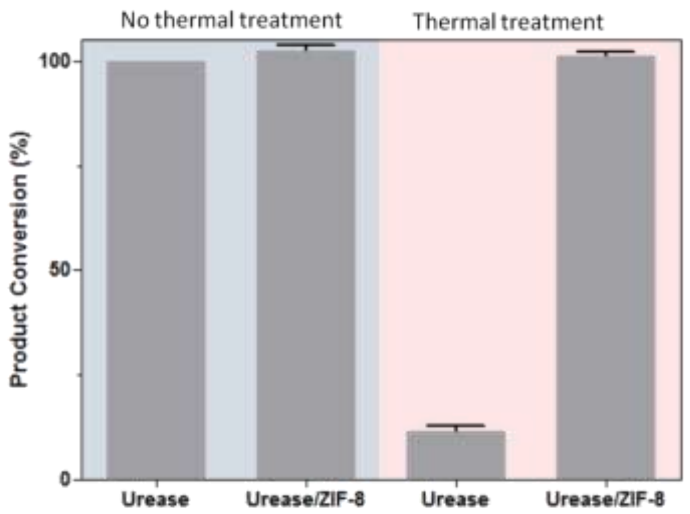


| | Urease | Urease/ZIF-8 | Urease thermal treatment | Urease/ZIF-8 thermal treatment |
|---|---|---|---|---|
| Half time (min) | 40 | 17.1 | -- | 105 |
| $V_0$ (Abs min$^{-1}$) | $1.2x10^{-2}$ | $2.12x10^{-2}$ | $1.0x10^{-7}$ | $5.66x10^{-3}$ |

**Supplementary Figure 22**. Normalized product conversion of ZIF-8/urease versus free urease before and after thermal treatment in 80 °C water for 1 h. Experiments were performed in triplicate. The activity of urease was determined using phenol red as a pH indicator as a result of urea to ammonia conversion. Half time is the time to reach half of the maximum substrate conversion; $V_0$ is the initial substrate conversion rate of the enzyme.

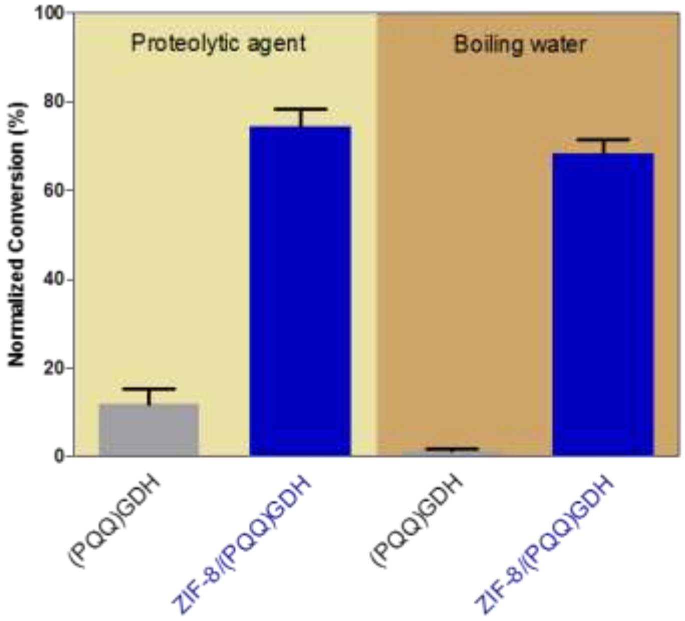


**Supplementary Figure 23**. Normalized product conversion of ZIF-8/(PQQ)GDH versus free (PQQ)GDH in the presence of proteolytic agent and after the treatment in boiling water. The activity of (PQQ)GDH was determined using phenazine methosulfate as an electron acceptor.[11,12]

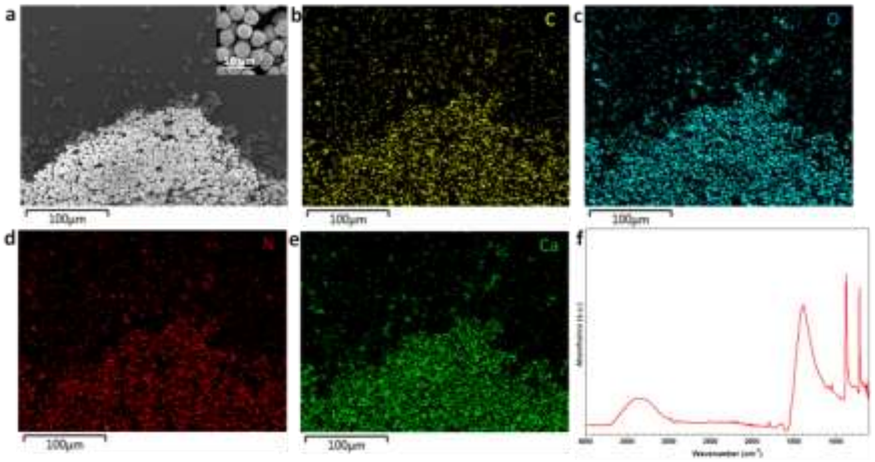


**Supplementary Figure 24**. a) SEM and b-e) EDX images of HRP-loaded $CaCO_3$ particles. f) FTIR spectra of HRP-loaded $CaCO_3$ particles. Peaks at 711, 874, and ~1400 $nm^{-1}$ are characteristic of $CaCO_3$,[13,14] the amide I (1655 $cm^{-1}$) and amide II (1548 $cm^{-1}$) peaks are characteristics of the enzymes[15,16]

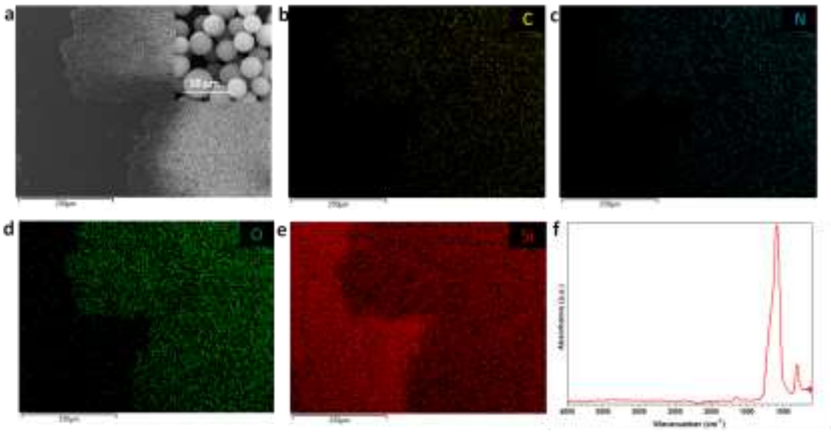

**Supplementary Figure 25**. a) SEM and b-e) EDX images of HRP-loaded $SiO_2$ (SGX) particles. f) FTIR spectra of HRP-loaded $SiO_2$ (SGX) particles. The presence of C and N elements in the EDX results confirmed the presence of HRP. The 1111 $cm^{-1}$ peak with a shoulder at 1188 $cm^{-1}$ is assigned to the transverse-optic and longitudinal-optic modes of the Si-O-Si asymmetric stretching vibrations, while the peak at 800 $cm^{-1}$ can be assigned to Si-O-Si symmetric stretching vibrations.[17] The peak at 1655 $cm^{-1}$ is assigned to the amide I bond characteristic of the enzymes[15,16]

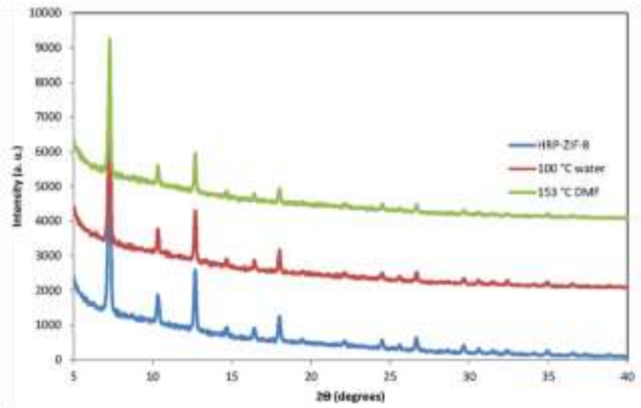


**Supplementary Figure 26.** PXRD measurements of HRP-biomimetically mineralized ZIF-8 (blue) before, and after the treatment in boiling water (red) and boiling DMF (green), respectively.

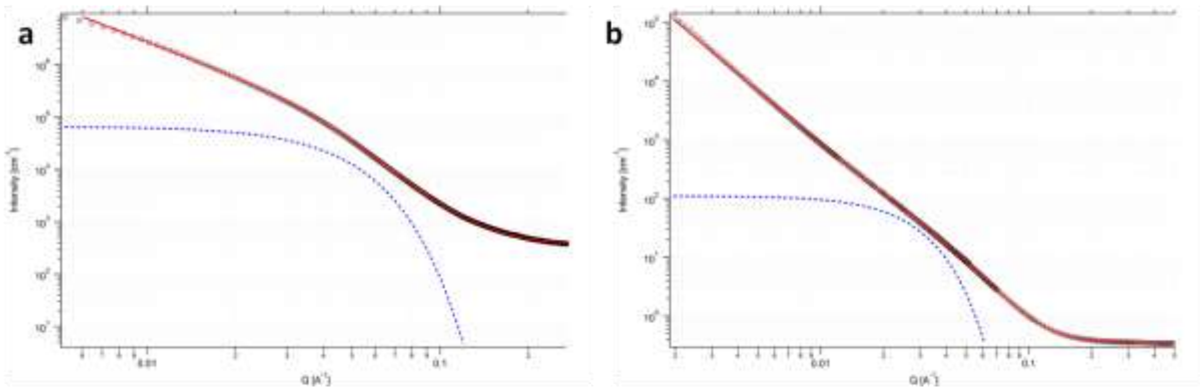


**Supplementary Figure 27**. SAXS plot of the intensity (counts) versus q($Å^{-1}$) of (a) ZIF-8/HRP and (b) ZIF-8/Urease. Unified fit (red) of experimental data (black), Guinier component (blue) of unified fit showing the presence of new generation of pores within ZIF-8 with Rg = 45 Å for ZIF-8/HRP and Rg = 68 Å for ZIF-8/urease. These pore sizes are found to be 28% and 30% larger than that of HRP[18,19] and Urease[20], respectively.

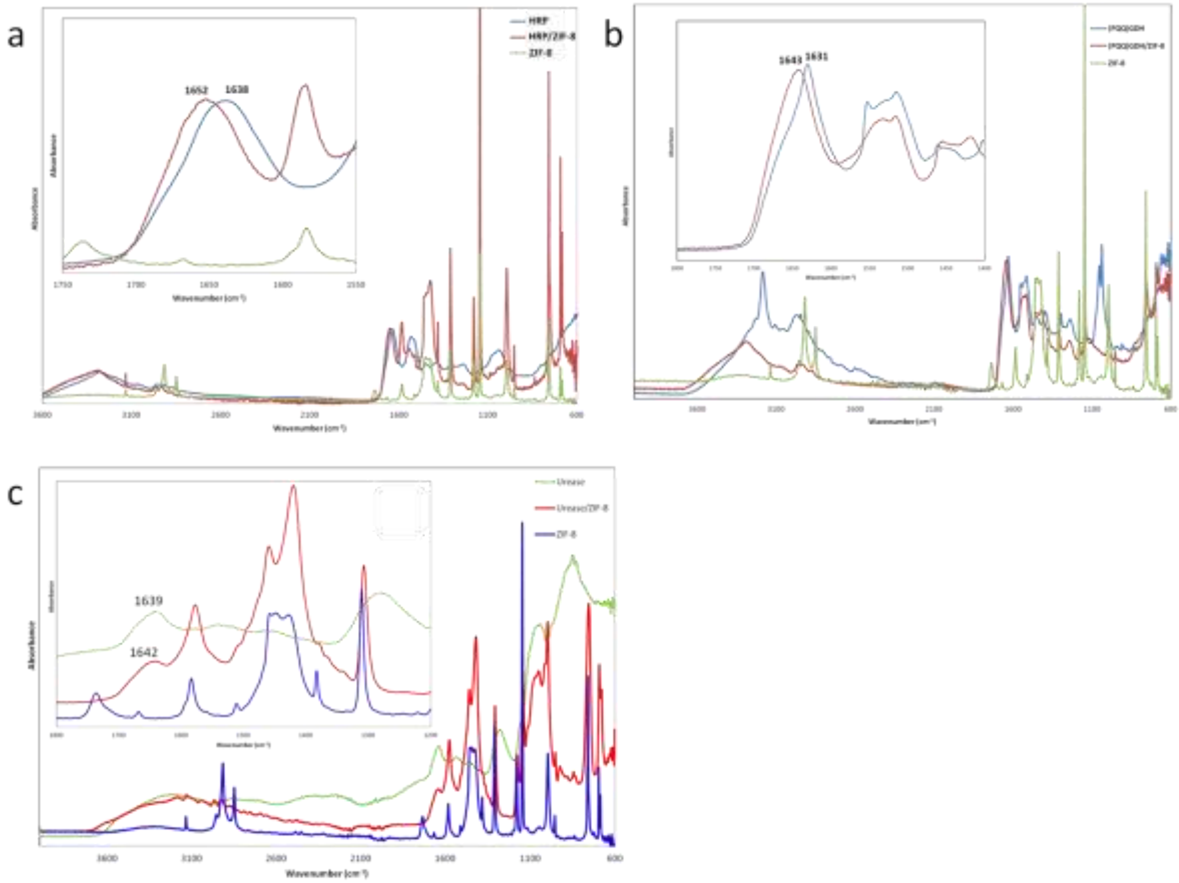


**Supplementary Figure 28**. FTIR spectra of (a) HRP (blue), HRP-biomimetically mineralized ZIF-8 (red), and standard ZIF-8 (green); (b) (PQQ)GDH (blue), (PQQ)GDH-biomimetically mineralized ZIF-8 (red), and standard ZIF-8 (green); (c) urease (blue), urease-biomimetically mineralized ZIF-8 (red), and standard ZIF-8 (green). Insets show the zoomed-in spectra. In all the biomimetically mineralized ZIF-8 samples, the amide I peak from the enzymes (mainly from C=O stretching mode) showed a shift towards higher wavenumbers, indicating the interaction between the carbonyl groups of the protein backbone and the $Zn^{2+}$ cations of ZIF-8.[8]

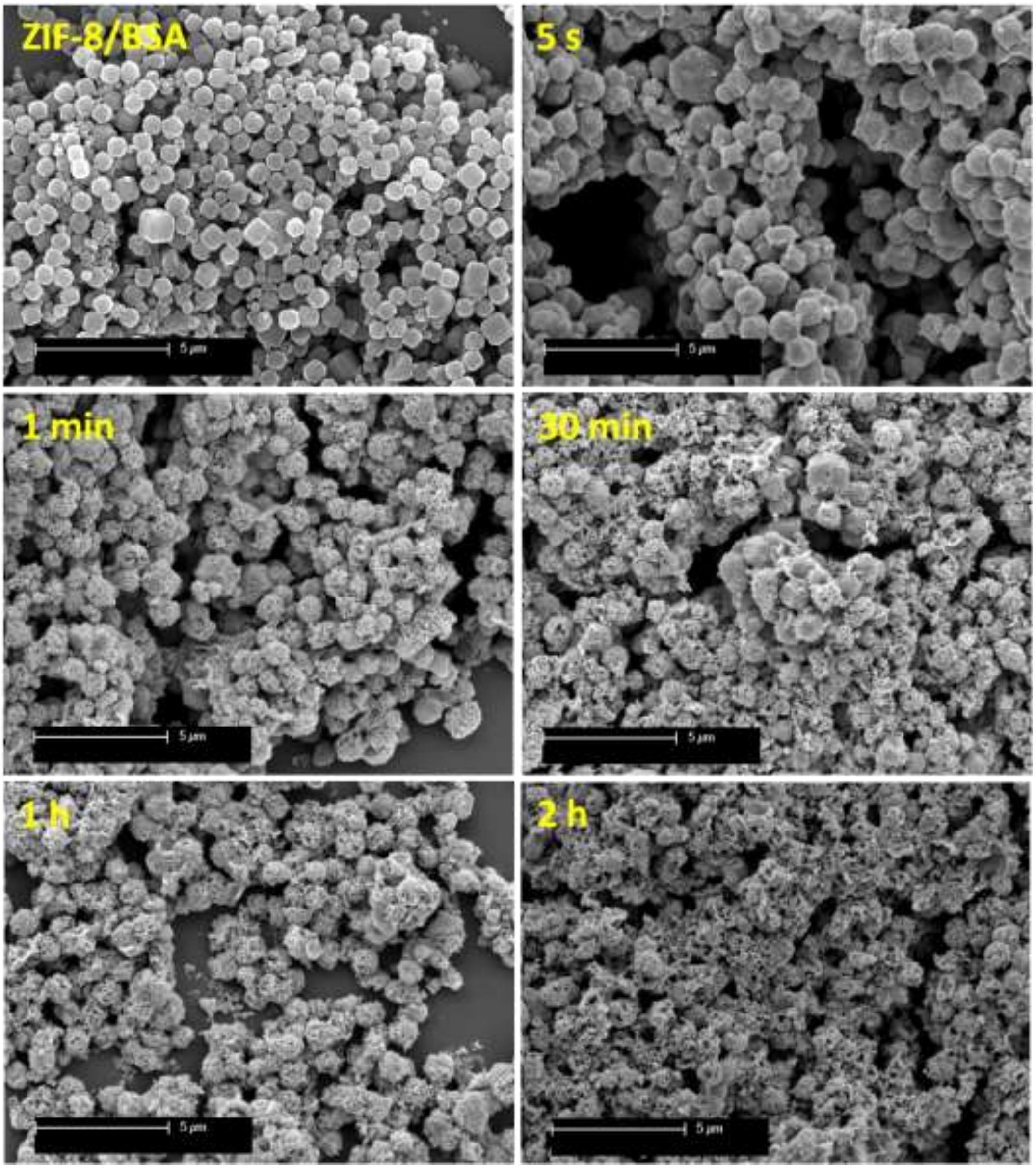


**Supplementary Figure 29.** SEM images showing the progressive decomposition of ZIF-8/BSA crystals at pH 6.0 over time.

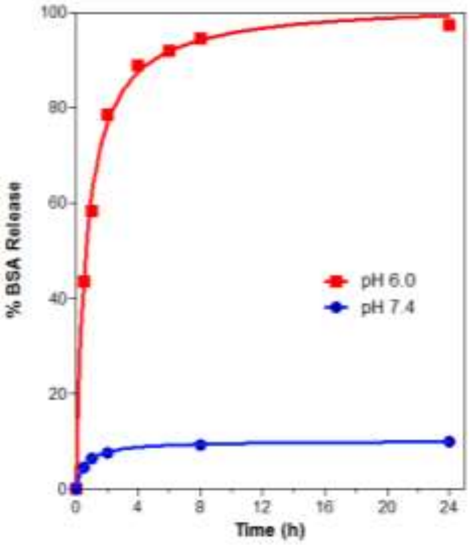


**Supplementary Figure 30.** Release of FITC-labelled BSA from ZIF-8/BSA biocomposites in PBS at pH 6.0 and 7.4, assessed using fluorescence spectrophotometry.

## Supplementary Methods

**Preparation of standard ZIF-8.** 2-methylimidazole (160 mM, 20 mL) was dispersed in methanol at room temperature. A separate solution of zinc acetate dissolved in methanol (40 mM, 20 mL) was also prepared. These two solutions were combined and then agitated for 10 s and aged for 24 h to grow ZIF-8 crystals. Crystals with typical rhombic dodecahedron morphology were collected and washed 3 times with fresh methanol. This sample was used as a standard for a comparison with the biomimetically mineralized ZIF-8.

**Fluorescent labelling of proteins** 1 mg of fluorescein isothiocyanate (FITC) and 35 mg of BSA were dissolved in 2.5 mL 3-(N-morpholino)propanesulfonic acid (MOPS) (10 mM, pH 7.0) buffer and left for 2 h at room temperature undergentle agitation. The FITC-labelled BSA was recovered by passing the mixture through an Illustra NAP-25 column (GE Healthcare Life sciences, NSW, Australia).

**Synthesis of $CaCO_3$ particles** enzyme-loaded $CaCO_3$ particles with 30-50 nm pores were synthesized according to previously-reported methods.[21,22] The $Na_2CO_3$ (330 mM in deionised water) solution was rapidly mixed with equal volumes of the $CaCl_2$ (330 mM) solution containing HRP (2 mg/mL in deionised water) followed by vigorous stirring for 30 s at room temperature. The solution was then aged for 15 min without stirring. The obtained precipitate was recovered by centrifugation at 1000 g for 2 min in water. The encapsulation efficiency (28%) of HRP in $CaCO_3$ particles was determined using UV-Vis spectroscopy at 280 nm from a pre-determined calibration curve, by measuring the concentrations of the HRP in the precursor solution and in the supernatant of the obtained particles.

**Enzyme immobilization on SGX silica particles** For the preparation of HRP-loaded silica particles, the surfaces of the silica particles were modified with aminopropyltriethoxysilane (APTES). 10 mg silica particles (average pore size 7 nm SBA-15, ACS Material, LLC; 20, 50,

and 100 nm pore size, TESSEK Ltd.) were suspended in toluene (5 mL) including 0.5 mL APTES. After stirring for 12 h at room temperature, the APTES-modified silica particles were washed with ethanol and water in consecutive washing/centrifugation cycles for three times and finally dispersed in MES buffer (1 mL, 0.1 M, pH 5). HRP (1 mg) and 1-ethyl-3-(3-dimethylaminopropyl)carbodiimide hydrochloride (EDC, 1 mg) was then introduced into the silica particle suspension and incubated for 2 h under constant gentle agitation. Enzyme loading effieiency: 82.0% (7 nm pore $SiO_2$), 68.8% (20 nm pore $SiO_2$), 69.1% (50 nm pore $SiO_2$), 66.2% (100 nm pore $SiO_2$).

**Characterization techniques**

**BET** surface areas of ZIF-8 and the MOF bio-composites were determined using nitrogen sorption at -196°C using a Micromeritics 6 port ASAP 2420 analyser. The samples were degassed at 120°C for 8 hours under vacuum prior to analysis. The pore size distribution was determined using the Density Functional Theory (DFT) of capillary condensation at -196°C for $N_2$ in cylindrical pores. The DFT model for capillary condensation has been shown to be suitable for reversible and hysteretic isotherms of nitrogen on cylindrical, spherical and slit-like materials.[23–25] The DFT model treats each pore independently and accounts for non-homogeneous molecular interactions.[26,27] Since 1989, this model has been used to determine the pore size distribution from adsorpion isotherms.[28] DFT is a commonly used method for determining the pore size distribution in MOFs and in particular ZIF-8.[29–32] In our work the silica/zeolite potentials were utilized to describe the molecular interactions.

The shape of the adsorption isotherm is affected by the energetic heterogeneity and geometric topology of the samples and usually features a distribution of pore sizes. DFT works on the theory that the isotherm is the convolution of the adsorption process of several properties

which affect the adsorption process.[27] Therefore the integral equation of adsorption of these surface energy distributions can be expressed as[27]

$$Q(p) = \int da\, db\, dc \ldots q(p, a, b, c \ldots) f(a, b, c \ldots) \qquad (1)$$

Where

Q(p) = the total quantity adsorbed per unit weight at pressure p,

a,b,c…= a set of distributed properties

f(a,b,c…) = the distribution function of the properties and

q(p,a,b,c,…) = the kernel function describing the adsorption isotherm on unite surface of material with fixed properties a,b,c…

In order to solve the above expression, the integral equation of adsorption can be characterised by a distribution of adsorptive energies;[27]

$$Q(p) = \int d\varepsilon\, q(p, \varepsilon)\, f(\varepsilon) \qquad (2)$$

Where

Q(p) = the experimental quantity adsorbed per gram at pressure p,

q(p,e) = the quantity adsorbed per unit area at the same pressure, p, on an ideal free surface of energy e, and

f(e) = the total area of surface of energy e in the sample.

Therefore, assuming that each pore acts independently, its distribution of pore sizes can be determined by[27]

$$Q(p) = \int dH\, q(p, H)\, f(H) \qquad (3)$$

Where

Q(p) = the experimental quantity adsorbed at pressure p,

q(p,H) = the quantity adsorbed per unit area at the same pressure, p, in an ideal pore size H, and

f(H) = the total area of pores of size H in the sample.

The equations (2) and (3) are then integrated over all surface energies and pore sizes and solved in a discrete form;

$$Q(p) = \sum_i q(p, Z_i) f(Z_i) \qquad (4)$$

The micromeritics software then determines the set of positive f(Z) values that best fits equation 4 once given the appropriate model chosen from the available library (cylindrical fit using the silica/zeolite potentials).[27]

**Confocal Laser Scanning Microscopy** (CLSM) was used to determine the presence of dye labelled bio-molecules (e.g. BSA and oligonucleotides) within a Leica TCS SP5.

For **XRD Lab measurements**, the ZIF-8, Eu-BDC, Tb-BDC and $CaCO_3$ samples were loaded into glass capillaries and examined in Debye-Sherrer transmission geometry[33] using a PANalytical X'Pert PRO Multi-Purpose Diffractometer, employing Co $K_\alpha$ radiation. The incident beam was collimated using Soller slits, and a graphite post-diffraction monochromator (used to minimise the $K_\beta$ signal) was fitted in front of the detector. The sample capillaries were spun at ~2 Hz to improve the particle statistics. Data were collected between 5 and 65° 2θ for times ranging from 0.5 to 1.5 hours, with a step size of 0.03° 2θ. The acquired diffraction patterns were analysed by either the crystal structure-based Rietveld method,[34,35] or the Pawley method,[5] using the Topas-Academic software package (version 4.1,

Coelho Software, 2007). For these measurements the observed peak profiles were modelled using the Fundamental Parameters Approach.[36]

**Synchrotron XRD** data were collected from the ZIF-8 and HKUST-1 samples using the powder diffraction beamline at the Australian Synchrotron.[37] An X-ray beam energy of 16.82 keV ($\lambda$ = 0.736828 Å) was selected, and diffraction patterns were collected between 5 to 85.5° 2θ using the high-resolution Mythen detector.[38] The diffraction patterns were acquired in pairs in order to eliminate the gaps between the modules of the Mythen detector. These diffraction pattern pairs were merged using the program CONVAS2[39] and analysed using the Rietveld method. An empirical model for the instrument was derived based on data collected from a sample of $LaB_6$ (NIST SRM 660b) mixed with diamond powder.

For the purposes of comparison with existing literature, all the XRD data presented here have been scaled to the wavelength of Cu Kα radiation ($\lambda$ = 1.54059 Å).

**Synchrotron SAXS** data were collected at the SAXS beamline of the Australian Synchrotron facility.[40] Capillaries were loaded with the washed and dried sample. The samples were investigated using the SAXS/WAXS beamline (9.3 keV, 2675 mm camera length using a Pilatus 1M as detector, transmission mode). For each SAXS analysis, 4 measurements (different positions) were averaged for each capillary, and the background of an empty capillary was subtracted. Scatterbrain software was used for both the averaging and the background subtraction process.

The size (radius of gyration, $R_g$) of new generation of pores hosting biomacromolecules in the biomimetically mineralized ZIF-8 was measured at the Australian Synchrotron SAXS/WAXS beamline. Results were characterised using Guinier knee fitting using the Unified model. Beaucage[1] describes how Guinier's law and structurally limited power laws

can be derived from mutually exclusive scattering events. In the simplest case, the observed scattering is a summation of two components,

$$I(q) \approx G \exp\left(\frac{-q^2 R_g^2}{3}\right) + B \left[\frac{\left(\mathrm{erf}\left(\frac{qR_g}{\sqrt{6}}\right)\right)^3}{q}\right]^P, \qquad (5)$$

where G is the classic Guinier prefactor and B is a prefactor specific to the type of power-law scattering, specified by the regime in which the exponent P, falls. The momentum transfer, q, has the units $(\text{length})^{-1}$ so large q scattering probes small lengthscales. For a surface fractal,

$$B=4\pi^2\rho^2 R_g^{(6-P)}\tau((P-1)\sin(\pi(P-3)/2)(P-3)), \qquad (6)$$

where $R_g$ is the large particulate radius of gyration. The error function (eft) is available in a number of fitting programs (e,g, Igor) or can be calculated using an asymptotic expansion.[41]

**Scanning electron microscopy** (SEM) was used to investigate the particle morphology using a Philips XL30 Field Emission Scanning Electron Microscope (FESEM) equipped with an Energy Dispersive X-ray detector (EDS, Oxford Instruments).

**FTIR** was performed using an alpha Bruker spectrometer using both, transmission and ATR modes (128 scans, 2 $cm^{-1}$ resolution).

**Fluorescence spectrophotometry** Fluorescence measurements of solutions were carried out using a Fluorolog-3 Model FL3-22 spectrofluorometer (Jobin Yvon Inc., USA) equipped with a HgXe lamp.

**UV-Vis** absorption spectra were collected using a single beam spectrometer model SpectroVis Plus (Vernier Software & Technology, Beaverton OR, USA).

**ICP** analyses were performed using a Varian ICP-OES spectrometer model 730-ES, operating in simultaneous wavelengths scanning. The ion concentrations were calculated from comparison with a suitable calibration curve.

**$^1$H-NMR** experiments were conducted on a Bruker BioSpin Avance$^{III}$ NMR spectrometer operating at 500 MHz (11.7 T magnet), using $D_2O$ as solvent.

## Supplementary References


1. Beaucage, G. Small-Angle Scattering from Polymeric Mass Fractals of Arbitrary Mass-Fractal Dimension. *J. Appl. Crystallogr.* **29,** 134–146 (1996).
2. Wilkins, D. K. *et al.* Hydrodynamic Radii of Native and Denatured Proteins Measured by Pulse Field Gradient NMR Techniques†. *Biochemistry (Mosc.)* **38,** 16424–16431 (1999).
3. Mylonas, E. & Svergun, D. I. Accuracy of molecular mass determination of proteins in solution by small-angle X-ray scattering. *Journal of Applied Crystallography* (2007).
4. Daiguebonne, C. *et al.* Structural and Luminescent Properties of Micro- and Nanosized Particles of Lanthanide Terephthalate Coordination Polymers. *Inorg. Chem.* **47,** 3700–3708 (2008).
5. Pawley, G. S. Unit-cell refinement from powder diffraction scans. *J. Appl. Crystallogr.* **14,** 357–361 (1981).
6. Nevskaya, N. A. & Chirgadze, Y. N. Infrared spectra and resonance interactions of amide-I and II vibration of alpha-helix. *Biopolymers* **15,** 637–648 (1976).
7. Byler, D. M. & Susi, H. Examination of the secondary structure of proteins by deconvolved FTIR spectra. *Biopolymers* **25,** 469–487 (1986).
8. Feng, Y., Schmidt, A. & Weiss, R. A. Compatibilization of Polymer Blends by Complexation. 1. Spectroscopic Characterization of Ion−Amide Interactions in Ionomer/Polyamide Blends. *Macromolecules* **29,** 3909–3917 (1996).
9. Tajmir-Riahi, H. A. & Ahmed, A. Complexation of copper and zinc ions with proteins of a light-harvesting complex (LHC-II) of chloroplast thylakoid membranes studied by FT-IR spectroscopy. *J. Mol. Struct.* **297,** 103–108 (1993).
10. Wang, X., Zhou, J., Tong, P. S. & Mao, X. Y. Zinc-binding capacity of yak casein hydrolysate and the zinc-releasing characteristics of casein hydrolysate-zinc complexes. *J. Dairy Sci.* **94,** 2731–2740 (2011).
11. Okuda, J. & Sode, K. PQQ glucose dehydrogenase with novel electron transfer ability. *Biochem. Biophys. Res. Commun.* **314,** 793–797 (2004).
12. Sode, K. *et al.* Over expression of PQQ glucose dehydrogenase in Escherichia coli under holo enzyme forming condition. *Biotechnol. Lett.* **16,** 1265–1268 (1994).
13. Politi, Y., Arad, T., Klein, E., Weiner, S. & Addadi, L. Sea Urchin Spine Calcite Forms via a Transient Amorphous Calcium Carbonate Phase. *Science* **306,** 1161–1164 (2004).
14. Gago-Duport, L., Briones, M. J. I., Rodríguez, J. B. & Covelo, B. Amorphous calcium carbonate biomineralization in the earthworm's calciferous gland: Pathways to the formation of crystalline phases. *J. Struct. Biol.* **162,** 422–435 (2008).
15. Barth, A. & Zscherp, C. What vibrations tell us about proteins. *Q. Rev. Biophys.* **35,** 369–430 (2002).
16. Reiter, G., Hassler, N., Weber, V., Falkenhagen, D. & Fringeli, U. P. In situ FTIR ATR spectroscopic study of the interaction of immobilized human tumor necrosis factor-α with a

monoclonal antibody in aqueous environment. *Biochim. Biophys. Acta BBA - Proteins Proteomics* **1699,** 253–261 (2004).
17. Musić, S., Filipović-Vinceković, N. & Sekovanić, L. Precipitation of amorphous SiO2 particles and their properties. *Braz. J. Chem. Eng.* **28,** 89–94 (2011).
18. Di Fabrizio, E. *et al.* Microlithographic techniques for laser assisted fabrication of bioelectronic devices. *Appl. Phys. Lett.* **69,** 3280–3282 (1996).
19. Joo, H. & Yoo, Y. J. Determination of Horseradish Peroxidase Structure-Activity Relationships by Measuring Radius of Gyration in Water-Miscible Organic Solvents. *Throries Appl. Chem. Eng.* **1,** 475 (1995).
20. Hirai, M., Kawai-Hirai, R., Hirai, T. & Ueki, T. Structural change of jack bean urease induced by addition surfactants studied with synchrotron-radiation small-angle X-ray scattering. *Eur. J. Biochem.* **215,** 55–61 (1993).
21. Volodkin, D. V., Larionova, N. I. & Sukhorukov, G. B. Protein Encapsulation via Porous CaCO3 Microparticles Templating. *Biomacromolecules* **5,** 1962–1972 (2004).
22. Petrov, A. I., Volodkin, D. V. & Sukhorukov, G. B. Protein—Calcium Carbonate Coprecipitation: A Tool for Protein Encapsulation. *Biotechnol. Prog.* **21,** 918–925 (2005).
23. Ravikovitch, P. I., Domhnaill, S. C. O., Neimark, A. V., Schueth, F. & Unger, K. K. Capillary Hysteresis in Nanopores: Theoretical and Experimental Studies of Nitrogen Adsorption on MCM-41. *Langmuir* **11,** 4765–4772 (1995).
24. Ravikovitch, P. I. & Neimark, A. V. Characterization of nanoporous materials from adsorption and desorption isotherms. *Colloids Surf. Physicochem. Eng. Asp.* **187–188,** 11–21 (2001).
25. Ravikovitch, P. I. & Neimark, A. V. Density Functional Theory of Adsorption in Spherical Cavities and Pore Size Characterization of Templated Nanoporous Silicas with Cubic and Three-Dimensional Hexagonal Structures. *Langmuir* **18,** 1550–1560 (2002).
26. Neimark, A. V. The Method of Indeterminate Lagrange Multipliers in Nonlocal Density Functional Theory. *Langmuir* **11,** 4183–4184 (1995).
27. Micromeritics ASAP 2420 Operator's Manual. *Append. C* (2007).
28. Landers, J., Gor, G. Y. & Neimark, A. V. Density functional theory methods for characterization of porous materials. *Colloids Surf. Physicochem. Eng. Asp.* **437,** 3–32 (2013).
29. Liu, Y., Liu, H., Hu, Y. & Jiang, J. Development of a Density Functional Theory in Three-Dimensional Nanoconfined Space: H2 Storage in Metal−Organic Frameworks. *J. Phys. Chem. B* **113,** 12326–12331 (2009).
30. Song, Q. *et al.* Zeolitic imidazolate framework (ZIF-8) based polymer nanocomposite membranes for gas separation. *Energy Environ. Sci.* **5,** 8359–8369 (2012).
31. Zhao, D. *et al.* Iron imidazolate framework as precursor for electrocatalysts in polymer electrolyte membrane fuel cells. *Chem. Sci.* **3,** 3200–3205 (2012).
32. Liu, Y., Liu, H., Hu, Y. & Jiang, J. Density Functional Theory for Adsorption of Gas Mixtures in Metal−Organic Frameworks. *J. Phys. Chem. B* **114,** 2820–2827 (2010).
33. Klug, H. P. & Alexander, L. E. *X-ray diffraction procedures for polycrystalline and amorphous materials*. (Wiley, 1974).
34. Rietveld, H. M. A profile refinement method for nuclear and magnetic structures. *J. Appl. Crystallogr.* **2,** 65–71 (1969).
35. Young, R. A. *The Rietveld method*. **5.,** (International Union of Crystallograhy ;Oxford University Press, 1993).
36. Cheary, R. W., Coelho, A. A. & Cline, J. P. Fundamental Parameters Line Profile Fitting in Laboratory Diffractometers. *J. Res. Natl. Inst. Stand. Technol.* **109,** 1–25 (2004).
37. Wallwork, K. S., Kennedy, B. J. & Wang, D. The high resolution powder diffraction beamline for the Australian Synchrotron. in *AIP Conference Proceedings* (eds. Choi, J. & Rah, S.) **879,** 879–882 (Amer Inst Physics, 2007).
38. Bergamaschi, A. *et al.* The MYTHEN detector for X-ray powder diffraction experiments at the Swiss Light Source. *J. Synchrotron Radiat.* **17,** 653–668 (2010).
39. Rowles, M. R. CONVAS2: A program for the merging of diffraction data. *Powder Diffr.* **25,** 297–301 (2010).

40. Kirby, N. M. *et al.* A low-background-intensity focusing small-angle X-ray scattering undulator beamline. *J. Appl. Crystallogr.* **46,** 1670–1680 (2013).
41. Beaucage, G. & Schaefer, D. W. Structural studies of complex systems using small-angle scattering: a unified Guinier/power-law approach. *J. Non-Cryst. Solids* **172–174, Part 2,** 797–805 (1994).
